# Supplementary material for: Optical generative models
Source: Nature. 2025 Aug 27;644(8078):903–11. doi: 10.1038/s41586-025-09446-5 (PMC12390839; doi:10.1038/s41586-025-09446-5)
Supplement: Supplementary file 1 — This file includes details of the digital encoder, background information and definitions of DDPM, training details, Supplementary Figs. 1–27 and Table 1. [file 41586_2025_9446_MOESM1_ESM.pdf]

nature portfolio

https://doi.org/10.1038/s41586-025-09446-5

Supplementary information

Optical generative models

In the format provided by the authors and unedited

Supplementary Information for

Optical Generative Models

Shiqi Chen[a,b,c], Yuhang Li[a,b,c], Yuntian Wang[a,b,c], Hanlong Chen[a,b,c], Aydogan Ozcan[a,b,c*]
[a]Electrical and Computer Engineering Department, University of California, Los Angeles, CA, 90095, USA
[b]Bioengineering Department, University of California, Los Angeles, CA, 90095, USA
[c]California NanoSystems Institute (CNSI), University of California, Los Angeles, CA, 90095, USA
Corresponding to: ozcan@ucla.edu

This Supplementary Information file includes:

- Details of the Digital Encoder
- Background Information and Definitions of DDPM
- Training Details
- Supplementary Figures S1-S27
- Supplementary Table S1
- Supplementary Videos 1-9

1 Details of the Digital Encoder

For lower-resolution optical image generation, we use a variant of the multi-layer perceptron as the digital encoder. For a random sampled input $\mathcal{I}(x,y)\sim\mathcal{N}(0,I)$, the digital signal processed by the l_d^{th} layer can be calculated by:

$$\mathcal{H}^{(l_d)} = \kappa\left(W^{(l_d)}\mathcal{H}^{(l_d-1)} + b^{(l_d)}\right) \quad (1),$$

where the $\mathcal{H}^{(l_d-1)}$ is the output of the $(l_d-1)^{th}$ layer, and $\mathcal{H}^{(0)} = flatten(\mathcal{I}(x,y))$ is the input of the first layer. $W^{(l_d)} \in \mathbb{R}^{m_{l_d}\times m_{l_{d-1}}}$ is the weight matrix, $b^{(l_d)} \in \mathbb{R}^{m_{l_d}}$ is the bias, and m_{l_d} is the number of neurons in the l_d^{th} layer. Note that the last layer predicts a scaling factor s, so the digital encoder's output $\mathcal{H}_{out}^{(L_d)} \in \mathbb{R}^{hw+1}$ is split into 1D output signal $\mathcal{H}^{(L_d)} \in \mathbb{R}^{hw}$ and $s \in \mathbb{R}^1$. Then, the 1D output signal $\mathcal{H}^{(L_d)} \in \mathbb{R}^{hw}$ is reshaped to the 2D dimension of $\mathbb{R}^{h\times w}$ (denoted as $reshape(\cdot)$), which can be represented by the operation that reshapes a vector $v \in \mathbb{R}^{hw}$ into a matrix $\tilde{\phi} \in \mathbb{R}^{h\times w}$:

$$\tilde{\phi}_{a,b} = v_{(a-1)\times w+b} \quad (2),$$

where $a = 1,2,\cdots,h$ is the row index, $b = 1,2,\cdots,w$ is the column index.

For higher-resolution optical image generation (*e.g.*, for artwork generation), we utilized a field propagation-based processing pipeline. As shown in **Supplementary Fig. S6**, the digital encoder here consists of three parts: noise feature processor, in silico field propagator, and complex field converter that is conducted by a shallow U-Net. In the noise feature processor, the latent noise and the embedded class information are concatenated and processed by FC layers. Similar to lower-resolution optical image generation, the FC layer outputs the latent image feature and the scaling factor. Then, the latent features are unsampled and processed by the convolutional layers to produce a complex field ψ_o with the desired image dimension, which can be formulated as follows:

$$\psi_o = upconv(reshape(split(FC(\mathcal{H}^{(0)})))) \quad (3),$$

where the $FC(\cdot)$ maps the input feature $\mathcal{H}^{(0)} \in \mathbb{R}^{xyc+l}$ into $\mathcal{H}^{(1)} \in \mathbb{R}^{xyc\cdot n_{xy}^2\cdot n_c}$, x,y are the spatial dimensions of the latent feature, c is the channel dimension of the latent feature, n_{xy} is the spatial compression factor, and n_c is the channel compression factor. The $split(\cdot)$ operation separates the latent image features and the scaling factor. The $reshape(\cdot)$ is the same operation as in Eq. S2. The $upconv(\cdot)$ is an up-convolutional architecture composed of $1/(n_{xy}^2\cdot n_c)$ sequential up-sampling and convolutional layers (with $64k_c$ channels), interleaved with $LeakyReLU(\cdot)$ activation functions with a slope of 0.2. After the noise feature processor, an in silico field propagation is conducted from the image plane to the SLM plane, aiming to let the digital encoder interpret the processing conducted by the diffractive decoder. The complex field ψ_o at the image plane was numerically backward-propagated through the trainable diffractive decoder ϕ^{l_0} to the SLM plane ψ_{SLM}. The virtual complex field at the SLM plane ψ_{SLM} is calculated by:

$$\psi_{SLM}(x,y) = \mathcal{P}_f^{d_{0,1}}\mathcal{P}_m^{\phi_{l_0}}\mathcal{P}_f^{d_{1,2}}\left(\psi_o(x,y)\right) \quad (4),$$

where $d_{0,1}$ and $d_{1,2}$ are the axial distance from the SLM plane to the decoding layer and from the decoding layer to the sensor plane, respectively. $\mathcal{P}_m^{\phi_{l_0}}$ is the decoding phase

modulation. Finally, the complex field is converted to the encoded optical random seeds ϕ_{SLM} on the SLM plane:

$$\phi_{SLM} = Unet(\psi_{SLM}) \quad (5).$$

The complex field converter is a U-Net model that has 3 down-sampling and 3 up-sampling stages, with channel dimensions of $32k_u$, $64k_u$, $128k_u$, respectively. Here k_u is the channel multiplying factor to control the complexity of the complex field converter. The down-sampling and up-sampling operations between each stage use convolutional and transposed convolutional layers to realize 2x down/up-sampling, respectively. The down/up-sampling stages with the same spatial dimension have skip connections. In each stage, the features are processed by 2 Res-Blocks[1]. Before/after the processing of U-Net, the input/output images are passed through a convolutional layer to get the desired channel dimensions. We used $LeakyReLU(\cdot)$ activation function after every convolutional layer, and the negative slope was set to 0.2.

As shown in **Extended Data Fig. 7**, we investigated the performance of the digital encoder under different numbers of trainable parameters. In our implementation, (x, y, c) equals $(80, 80, 4)$ for each model and the factors to control the model size are listed in **Table S1** below:

Table. S1 Digital encoder details for higher resolution optical image generation

Parameters	44M	85M	166M	348M	580M
n_{xy}, n_c, k_c, k_u	1/2,1/4,1,1	1/2,1/2,1,2	1,1/4,2,4	1,1/2,4,8	1,1/2,8,24

2 Background Information and Definitions of DDPM

2.1 Background definitions

Let's take the notation that:

- The original image sampled from a target dataset is $\mathcal{I}_0$, and the diffusion process will add noise to it and gradually get $\mathcal{I}_1, \mathcal{I}_2, \cdots, \mathcal{I}_T$;
- $\mathcal{I}_t \sim q(\mathcal{I}_t)$ means $\mathcal{I}_t$ follows a probability distribution;
- $\mathcal{I}_T \sim \mathcal{N}(0, I)$, where I is the identity matrix;
- The noise added during the diffusion process follows the Gaussian distribution whose mean is 0 and variance is a hyperparameter $\beta_t \in (0,1)$. Each step of the diffusion process works based on the following equation:

$$q(\mathcal{I}_t|\mathcal{I}_{t-1}) = \mathcal{N}\left(\mathcal{I}_t; \sqrt{1-\beta_t}\mathcal{I}_{t-1}, \beta_t I\right) \quad (6),$$

- Because different step of the diffusion process follows the Markov Chain, we have,

$$q(\mathcal{I}_{1:T}|\mathcal{I}_0) = \prod_{t=1}^{T} q(\mathcal{I}_t|\mathcal{I}_{t-1}) \quad (7).$$

2.2 The forward process

In the forward noise sampling, Eq. S6 can be reparametrized as follows:

$$\mathcal{I}_t = \sqrt{1-\beta_t}\mathcal{I}_{t-1} + \sqrt{\beta_t}\epsilon_{t-1} \quad (8),$$

where $\epsilon_{t-1}\sim\mathcal{N}(0,I)$. Sequentially substituting $\mathcal{I}_{t-1}$ with the representation of $\mathcal{I}_{t-2}$, we can get:

$$\mathcal{I}_t = \sqrt{1-\beta_t}\left(\sqrt{1-\beta_{t-1}}\mathcal{I}_{t-2} + \sqrt{\beta_{t-1}}\epsilon_{t-2}\right) + \sqrt{\beta_t}\epsilon_{t-1}$$
$$= \sqrt{1-\beta_t}\left(\sqrt{1-\beta_{t-1}}\left(\sqrt{1-\beta_{t-2}}\mathcal{I}_{t-3} + \sqrt{\beta_{t-2}}\epsilon_{t-3}\right) + \sqrt{\beta_{t-1}}\epsilon_{t-2}\right) + \sqrt{\beta_t}\epsilon_{t-1}$$
$$= \cdots$$
$$= \sqrt{(1-\beta_t)(1-\beta_{t-1})\cdots(1-\beta_1)}\mathcal{I}_0 + \sqrt{(1-\beta_t)\cdots\beta_2\beta_1}\epsilon_0 + \cdots + \sqrt{(1-\beta_t)\beta_{t-1}}\epsilon_{t-2} + \sqrt{\beta_t}\epsilon_{t-1} \quad (9),$$

Because ϵ_t is a Gaussian distribution, if we take the notation: $\alpha_t = 1-\beta_t$, the ϵ_t term can be added up as a Gaussian:

$$\because \sqrt{1-\alpha_t}\epsilon_{t-1}\sim\mathcal{N}(0,(1-\alpha_t)I)$$
$$\sqrt{\alpha_t(1-\alpha_{t-1})}\epsilon_{t-2}\sim\mathcal{N}(0,\alpha_t(1-\alpha_{t-1})I)$$
$$\cdots$$
$$\sqrt{\alpha_t\cdots(1-\alpha_2)(1-\alpha_1)}\epsilon_0\sim\mathcal{N}(0,\alpha_t\cdots(1-\alpha_2)(1-\alpha_1)I)$$
$$\therefore \sqrt{(1-\beta_t)\cdots\beta_2\beta_1}\epsilon_0 + \cdots + \sqrt{(1-\beta_t)\beta_{t-1}}\epsilon_{t-2} + \sqrt{\beta_t}\epsilon_{t-1}\sim\mathcal{N}(0,(1-\alpha_t\alpha_{t-1}\cdots\alpha_1)I) \quad (10),$$

Therefore, the representation of $\mathcal{I}_t$ is:

$$\mathcal{I}_t = \sqrt{\alpha_t\alpha_{t-1}\cdots\alpha_1}\mathcal{I}_0 + \sqrt{1-\alpha_t\alpha_{t-1}\cdots\alpha_1}\epsilon \quad (11),$$

here $\epsilon\sim\mathcal{N}(0,I)$. Take the notation of $\bar{\alpha}_t = \prod_{s=1}^{t}\alpha_s$, Eq. S11 can be simplified as follows:

$$\mathcal{I}_t = \sqrt{\bar{\alpha}_t}\mathcal{I}_0 + \sqrt{1-\bar{\alpha}_t}\epsilon \quad (12),$$

Hence, Eq. S12 can be used in the forward sampling of the diffusion process, helping us directly get the noised sample at an arbitrary time step.

2.3 The reverse process

In the reverse process, the goal is to predict the approximate posterior probability $q(\mathcal{I}_{t-1}|\mathcal{I}_t)$. However, $q(\mathcal{I}_{t-1}|\mathcal{I}_t)$ cannot be calculated directly, and therefore, a model with learnable parameters was employed to predict its underlying distribution. With the prior information $\mathcal{I}_0$, $q(\mathcal{I}_{t-1}|\mathcal{I}_t,\mathcal{I}_0)$ can be formulated as:

$$q(\mathcal{I}_{t-1}|\mathcal{I}_t,\mathcal{I}_0) = q(\mathcal{I}_t|\mathcal{I}_{t-1},\mathcal{I}_0)\frac{q(\mathcal{I}_{t-1}|\mathcal{I}_0)}{q(\mathcal{I}_t|\mathcal{I}_0)} \quad (13),$$

here,

$$q(\mathcal{I}_t|\mathcal{I}_0)\sim\mathcal{N}(\bar{\alpha}_t\mathcal{I}_0,(1-\bar{\alpha}_t)\boldsymbol{I}) \quad (14),$$
$$q(\mathcal{I}_{t-1}|\mathcal{I}_0)\sim\mathcal{N}(\bar{\alpha}_{t-1}\mathcal{I}_0,(1-\bar{\alpha}_{t-1})\boldsymbol{I}) \quad (15),$$

Following the Eq. S12, we can get:

$$q(\mathcal{I}_t|\mathcal{I}_{t-1},\mathcal{I}_0) = q(\mathcal{I}_t|\mathcal{I}_{t-1})\sim\mathcal{N}(\bar{\alpha}_t\mathcal{I}_{t-1},(1-\bar{\alpha}_t)\boldsymbol{I}) \quad (16),$$

Using the definition of the Gaussian distribution:

$$\mathcal{N}(\mu,\sigma^2) \propto exp\left(-\frac{(x-\mu)^2}{2\sigma^2}\right) \quad (17),$$

$q(\mathcal{I}_{t-1}|\mathcal{I}_t,\mathcal{I}_0)$ can be expressed as:

$$q(\mathcal{I}_{t-1}|\mathcal{I}_t,\mathcal{I}_0) \propto exp\left(-\frac{1}{2}\left(\frac{\left(\mathcal{I}_t-\sqrt{\alpha_t}\mathcal{I}_{t-1}\right)^2}{\beta_t} + \frac{\left(\mathcal{I}_{t-1}-\sqrt{\bar{\alpha}_{t-1}}\mathcal{I}_0\right)^2}{1-\bar{\alpha}_{t-1}} - \frac{\left(\mathcal{I}_t-\sqrt{\bar{\alpha}_t}\mathcal{I}_0\right)^2}{1-\bar{\alpha}_t}\right)\right) \quad (18).$$

By simplifying this equation as the function of $\mathcal{I}_{t-1}$, the variance and the mean of

$q(\mathcal{I}_{t-1}|\mathcal{I}_t, \mathcal{I}_0)$ can be written as:

$$\begin{cases} \tilde{\sigma}_t{}^2 = \dfrac{1-\bar{\alpha}_{t-1}}{1-\bar{\alpha}_t} \cdot \beta_t \\ \tilde{\mu}_t = \dfrac{\sqrt{\alpha_t}(1-\bar{\alpha}_{t-1})}{1-\bar{\alpha}_t}\mathcal{I}_t + \dfrac{\sqrt{\bar{\alpha}_{t-1}}\beta_t}{1-\bar{\alpha}_t}\mathcal{I}_0 \end{cases} \quad (19).$$

Although $\mathcal{I}_0$ is unknown in the reverse process, it can be represented by $\mathcal{I}_t$ following the noise sampling equation in the forward procedure. Therefore, $q(\mathcal{I}_{t-1}|\mathcal{I}_t, \mathcal{I}_0)$ can be formulated as:

$$q(\mathcal{I}_{t-1}|\mathcal{I}_t) \sim q(\mathcal{I}_{t-1}|\mathcal{I}_t, \mathcal{I}_0) = \mathcal{N}\left(\frac{1}{\sqrt{\alpha_t}}\left(\mathcal{I}_t - \frac{1-\alpha_t}{\sqrt{1-\bar{\alpha}_t}}\epsilon_t\right), \left(\frac{1-\bar{\alpha}_{t-1}}{1-\bar{\alpha}_t}\cdot\beta_t\right)\boldsymbol{I}\right) \quad (20),$$

where ϵ_t is the noise of timestep t.

Therefore, the reverse process of the Denoising Diffusion Probabilistic Model (DDPM) is to gradually reconstruct the target data distribution by iteratively removing the noise ϵ_t and adding the perturbation from timestep t to $t-1$, which can be formulated as:

$$\mathcal{I}_{t-1} = \frac{1}{\sqrt{\alpha_t}}\left(\mathcal{I}_t - \frac{1-\alpha_t}{\sqrt{1-\bar{\alpha}_t}}\epsilon_t\right) + \sigma_t z \quad (21),$$

where $\bar{\alpha}_t = \prod_{s=1}^{t}\alpha_s$ and $\alpha_t = 1-\beta_t$, with $\beta_t = \beta_{start} + t/T \cdot (\beta_{end} - \beta_{start})$ being a linear function of the timestep $t \in [1, T]$. $\sigma_t{}^2 = (1-\bar{\alpha}_{t-1}/1-\bar{\alpha}_t)\cdot\beta_t$. $z \sim \mathcal{N}(0, I)$ for $t > 1$, $z = 0$ when $t = 1$. The DDPM uses a U-Net model to predict ϵ_t from the noised sample $\mathcal{I}_t$. Therefore, the objective of the U-Net is defined as follows:

$$\mathcal{L}(\theta_{U-Net}) = \min_{\theta_{U-Net}} E_{t\sim[1,T],\mathcal{I}_0\sim p_{data}(\mathcal{I}),\epsilon\sim\mathcal{N}(0,I)}\left[\left\|\epsilon_t - \epsilon_{\theta_{U-Net}}(\mathcal{I}_t, t)\right\|^2\right] \quad (22),$$

where θ_{U-Net} represents the parameters of the U-Net model, T is the total timestep in the denoising scheduler, $\mathcal{I}_0$ represents the original inputs sampled from the target data distribution, ϵ_t is the Gaussian noise sampled in each noising process, $\epsilon_{\theta_{U-Net}}(\mathcal{I}_t, t)$ is the operation to predict the additive noise according to the noised sample $\mathcal{I}_t$ and timestep t. For a given timestep t, the noised sample $\mathcal{I}_t$ is calculated by $\mathcal{I}_t = \sqrt{\bar{\alpha}_t}\mathcal{I}_0 + \sqrt{1-\bar{\alpha}_t}\epsilon_t$.

2.4 The coefficient of distribution transformation

The iterative optical generative model needs to minimize the expectation gap between the target distribution and the generated distribution. Therefore, the loss function in each timestep can be formulated as:

$$\mathcal{L}(\theta) = \min_{\theta_{model}} E_{t\sim[1,T],\mathcal{I}_0\sim p_{data}(\mathcal{I})}\left[\left\|m_{t-1,t} - \mathcal{O}_{\theta_{model}}(\mathcal{I}_t, t)\right\|^2\right] \quad (23),$$

here $m_{t-1,t} \sim \mathcal{N}\left(\frac{\mathcal{I}_t}{\sqrt{\alpha_t}}, \frac{1-\alpha_t}{\sqrt{\alpha_t(1-\bar{\alpha}_t)}}\boldsymbol{I}\right)$ is the distribution of the mean value in $q(\mathcal{I}_{t-1}|\mathcal{I}_t, \mathcal{I}_0)$, θ_{model} represents the parameters of the optical generative model, T is the total timestep in the denoising scheduler, $\mathcal{O}_{model}(\mathcal{I}_t, t)$ is the output of the optical generative model predicted from the noised sample $\mathcal{I}_t$ and timestep t. Because $\mathcal{I}_t$ can also be represented by the Gaussian process of $\mathcal{I}_0$ (Eq. S12), the expectation of $m_{t-1,t}$ can be formulated as:

$$E[m_{t-1,t}] \sim E\left[\mathcal{N}\left(\frac{\mathcal{I}_t}{\sqrt{\alpha_t}}, \frac{1-\alpha_t}{\sqrt{\alpha_t(1-\bar{\alpha}_t)}}\boldsymbol{I}\right)\right] = E\left[\mathcal{N}\left(\frac{\sqrt{\bar{\alpha}_t}\mathcal{I}_0 + \sqrt{1-\bar{\alpha}_t}\epsilon}{\sqrt{\alpha_t}}, \frac{1-\alpha_t}{\sqrt{\alpha_t(1-\bar{\alpha}_t)}}\boldsymbol{I}\right)\right] \quad (24).$$

Here $\epsilon \sim \mathcal{N}(0, 1)$ and therefore the coefficient of SNR_t can be simplified as $\sqrt{\bar{\alpha}_t}/\sqrt{\alpha_t}$ to

realize the transformation from the original data distribution $\mathcal{I}_0$ to the target data distribution $m_{t-1,t}$ during every t to $t-1$ transition.

2.5 Details of the U-Net model in the digital DDPM

The U-Net model has 3 down-sampling and 3 up-sampling stages, and the channel dimension of each stage is 224, 448, 672, respectively. The down-sampling and up-sampling operations between each stage use convolutional and transposed convolutional layers to realize 2x down/up sampling, respectively. The down/up sampling stages with the same spatial dimension have skip connections. In each stage, the features are processed by 2 Res-Blocks[1], where the timestep information is embedded as a vector and integrated with the features using the channel attention mechanism. For the embedding method of timesteps, we used the classic sinusoidal timestep embedding[2]. Before/after the processing of U-Net, the input/output images are passed through a convolutional layer to get the desired channel dimensions. We used $LeakyReLU(\cdot)$ activation function after every convolutional layer, and the negative slope was set to 0.2[3]. The details of the U-Net model are shown in **Supplementary Fig. S27.**

3 Training Details

The training was conducted on a server configured with an AMD Threadripper 3990X CPU, 128 GB of G.Skill DDR4 RAM (16 GB × 8), and four NVIDIA GeForce RTX 4090 GPUs. For training the teacher denoising diffusion probabilistic model (DDPM), we used a batch size of 200 and trained it for 300 epochs. The training time, which is a one-time effort, in GPU hours for each dataset is as follows: MNIST – 33 hours, Fashion-MNIST – 52 hours, Butterfly-100 – 48 hours, and CelebA – 58 hours. For fine-tuning the stable diffusion for artwork generation, we used a batch size of 8 and trained for 20 epochs, which took ~61 GPU hours. With more advanced GPUs, such as the NVIDIA A100, the training speed would be 3–8 times faster. This implies a reduction in the training time for the teacher DDPM, using a state-of-the-art GPU, down to approximately 6, 10, 9, and 11 GPU hours for MNIST, Fashion-MNIST, Butterfly-100, and CelebA, respectively.

For training the optical generative model, we used a batch size of 100 and trained for 100 epochs. In each iteration, we sampled a batch of random noise and fed it into the teacher model to obtain the data pair for training the optical generative model. We used 50 equally spaced steps of sampling to obtain the target distribution without significantly affecting the quality of the image generation. The training time of the optical generative model in GPU hours for each dataset is as follows: MNIST – 38 hours, Fashion-MNIST – 58 hours, Butterfly-100 – 59 hours, and CelebA – 70 hours. For training the optical generative model in artwork generation, we used a batch size of 4 and trained for 100 epochs (with 2500 iterations each epoch), which took approximately 82 GPU hours. Because the model we finetuned from stable diffusion shares the same latent representation for monochrome and multicolor image generation, the training time consumption is similar for these two tasks. Once again, with higher-end GPUs, such as the NVIDIA A100, the training speed would be 3–8 times faster. This implies a reduction in the

training time for the teacher DDPM to approximately 7, 11, 11, and 13 GPU hours for MNIST, Fashion-MNIST, Butterfly-100, and CelebA, respectively.

While training a deep learning model can be computationally intensive and time-consuming, it is generally a one-time process that occurs during the model development. Once the model is trained, it is fixed and reused across a wide range of deployment scenarios. In contrast, inference must be performed repeatedly—often in real-time or at large scale—during practical deployment. As a result, inference efficiency becomes much more critical for real-world utility, especially in applications requiring rapid response, low power consumption, or deployment on resource-constrained devices.

4 Supplementary Figures

Fig. S1: Diffractive phase structures of snapshot optical generative models

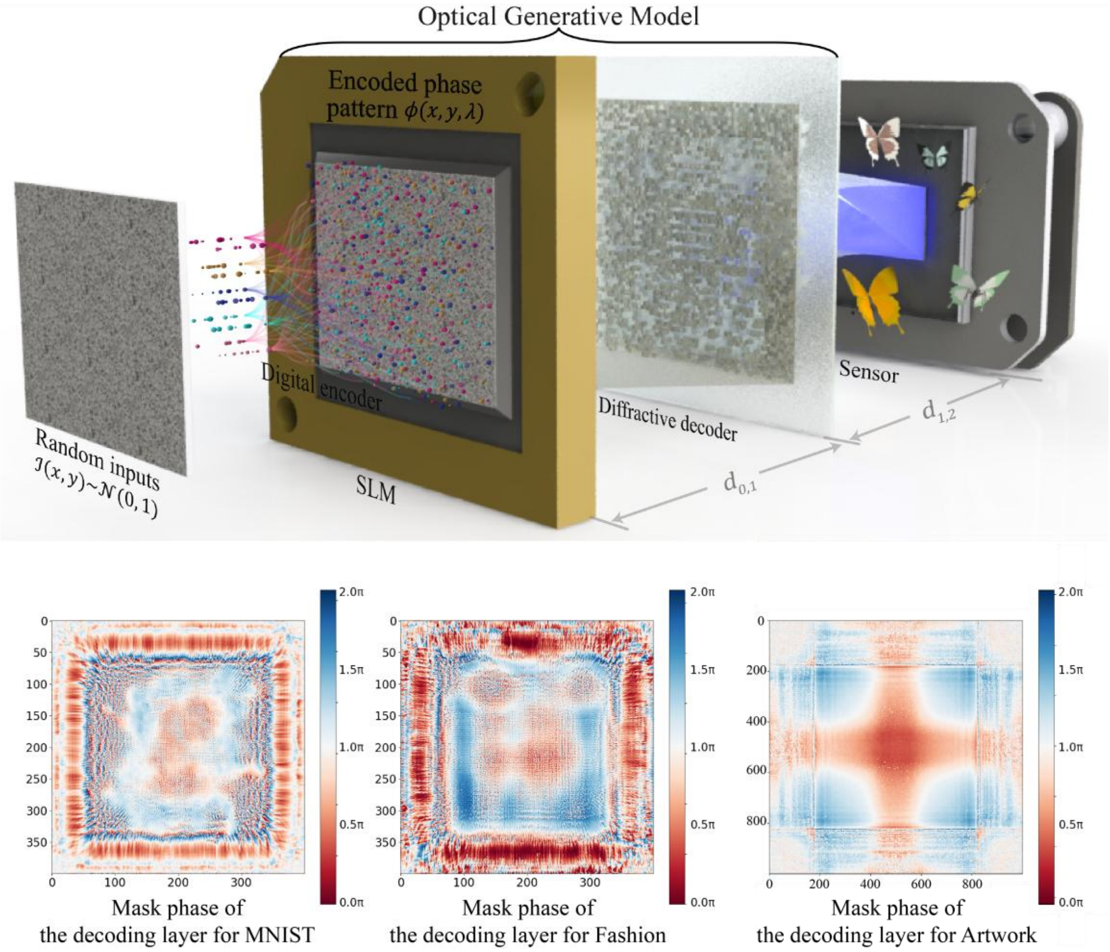

Snapshot optical generative model architecture and the fixed phase structures of the optimized decoding layers trained for generating novel images of MNIST, Fashion MNIST and Van Gogh-style Artworks.

Fig. S2: Experimental results of snapshot optical image generation of handwritten digits

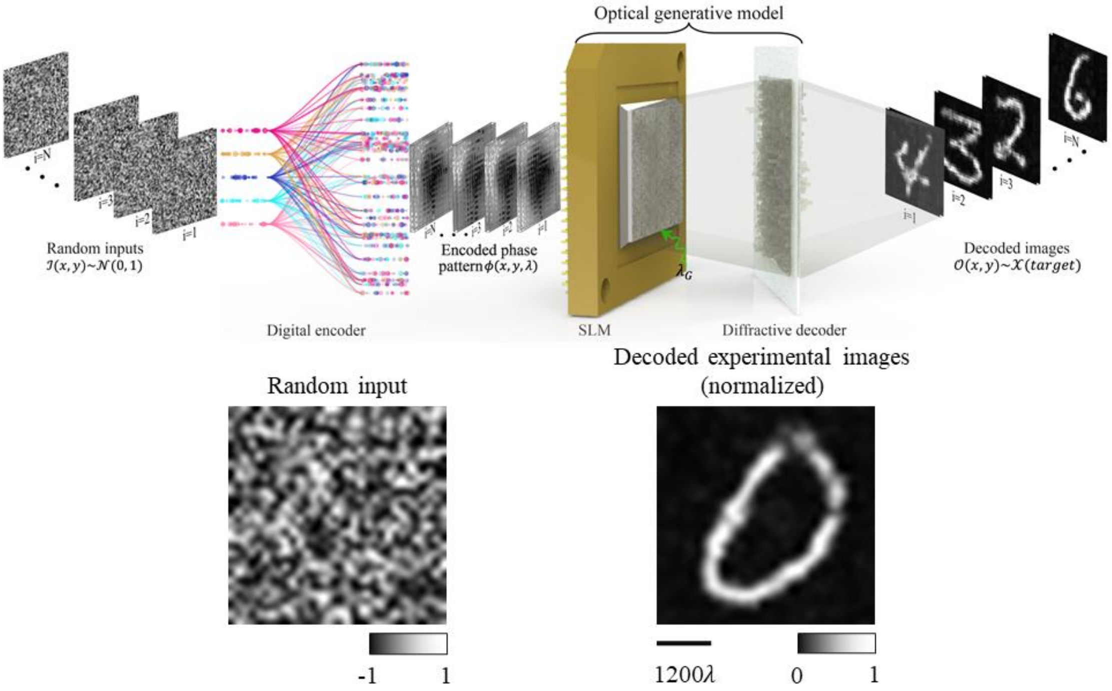

Optically generated novel images of handwritten digits. Please refer to **Supplementary Video 1** for details.

Fig. S3: Experimental results of snapshot optical image generation of fashion products

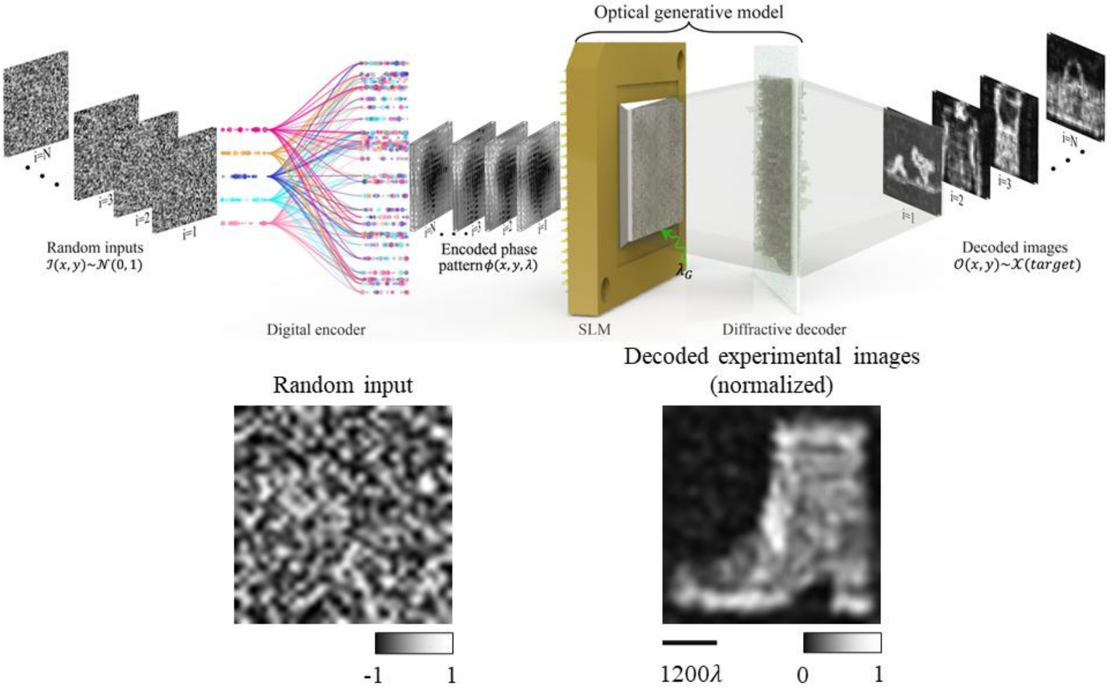

Optically generated novel images of fashion products. Please refer to **Supplementary Video 2** for details.

Fig. S4: Experimental demonstration of snapshot optical generative models with a limited phase range and decoder bit depth

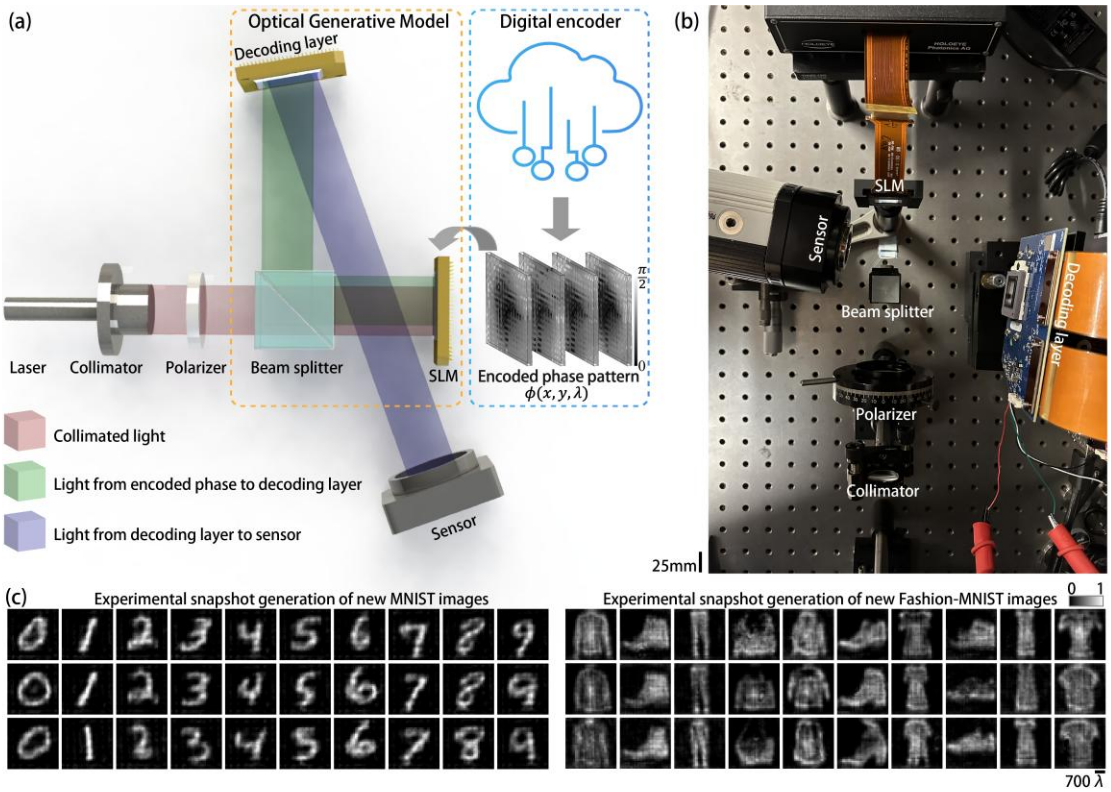

(a) The schematic of the snapshot optical generative model with a limited phase range and decoder bit depth, where the phase range of the optical random seed is limited to [0, $\pi/2$] and the phase bit depth of the decoding layer is limited to 16 depth levels (4 bits). (b) Photograph of the snapshot optical generative model. (c) The experimental results of novel image generation using the optical generative models trained for handwritten digits and fashion products, following the target data distributions of MNIST and Fashion-MNIST.

Fig. S5: Different views of the optical generative model set-up with a limited phase range and decoder bit depth used in Supplementary Fig. S15

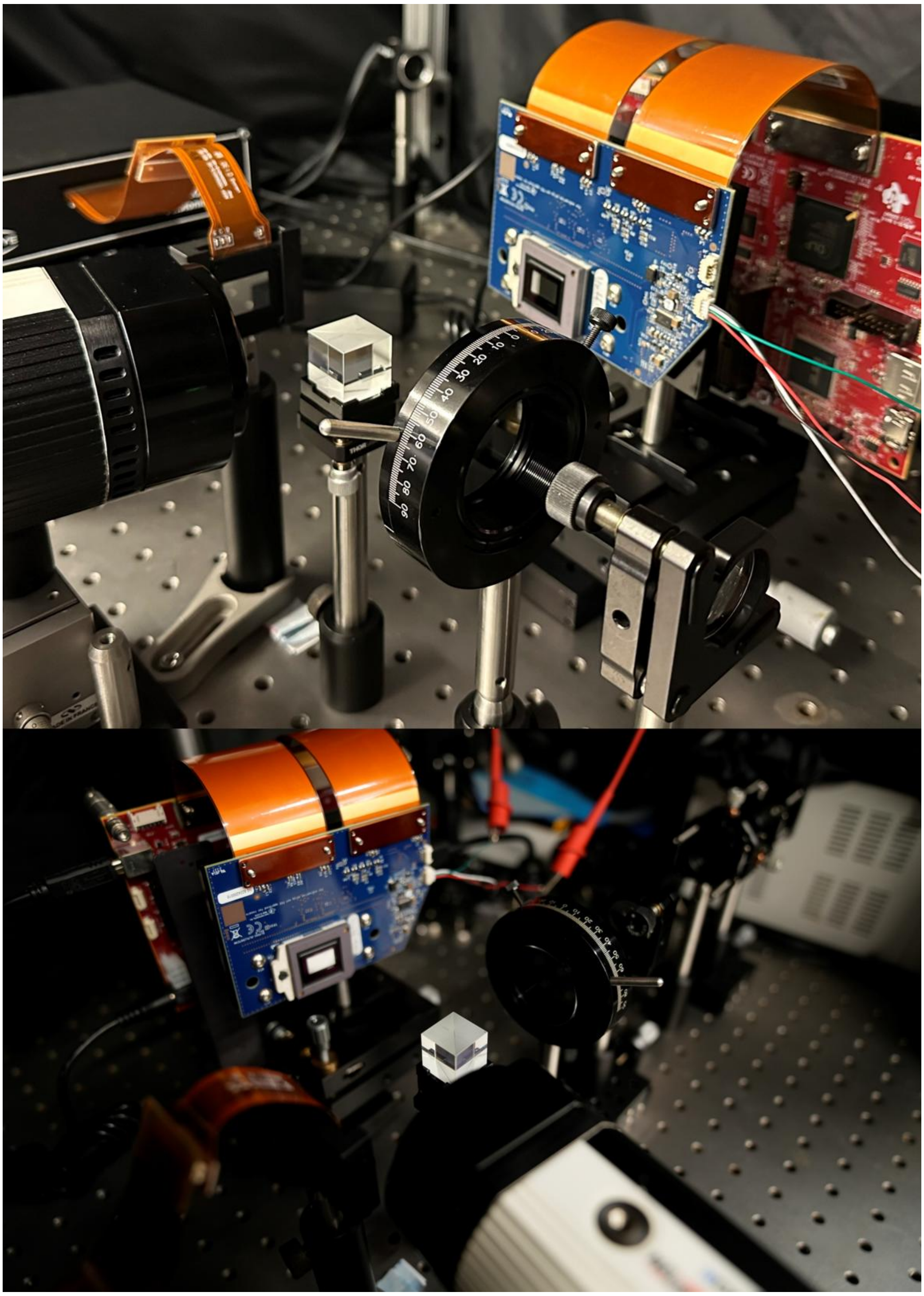

Fig. S6: The simulation pipeline for higher resolution snapshot optical image generation

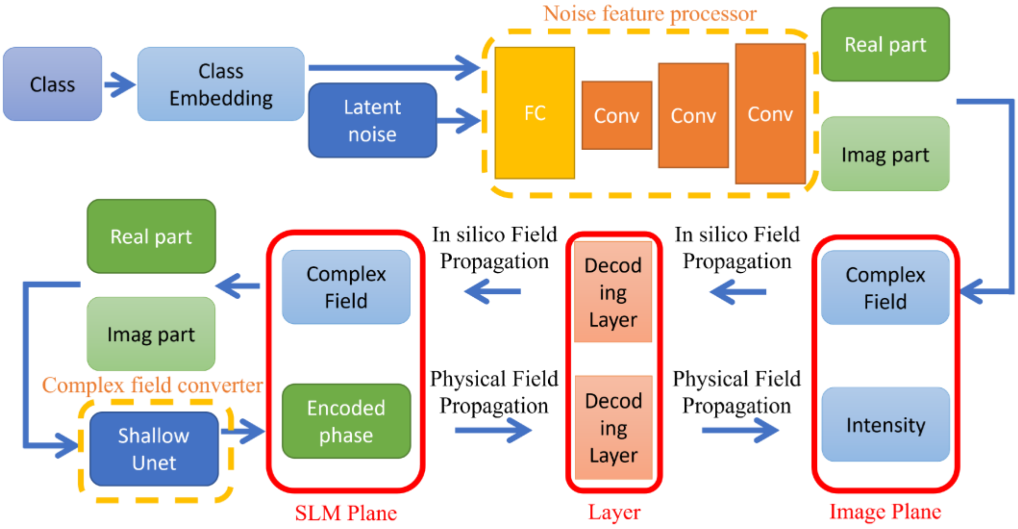

The higher-resolution optical image generation pipeline comprises a digital encoder and a physical field propagator. In the digital encoder, the latent noise and the embedded class information are concatenated and processed by FC layers. Then, the latent features are up-sampled by the transpose convolution layers. After that, an in silico field propagation from the image plane to the SLM plane is performed to obtain the complex field. The complex field on the SLM plane is then processed by a complex field converter to get the encoded phase pattern. Therefore, the digital encoder here consists of three main parts: noise feature processor, in silico field propagator, and complex field converter. The physical field propagator is implemented from the SLM plane to the image plane to simulate the optical image generation in the experimental set-up.

Fig. S7: Comparison of diffractive decoder and free-space decoder on Van Gogh style artwork generation.

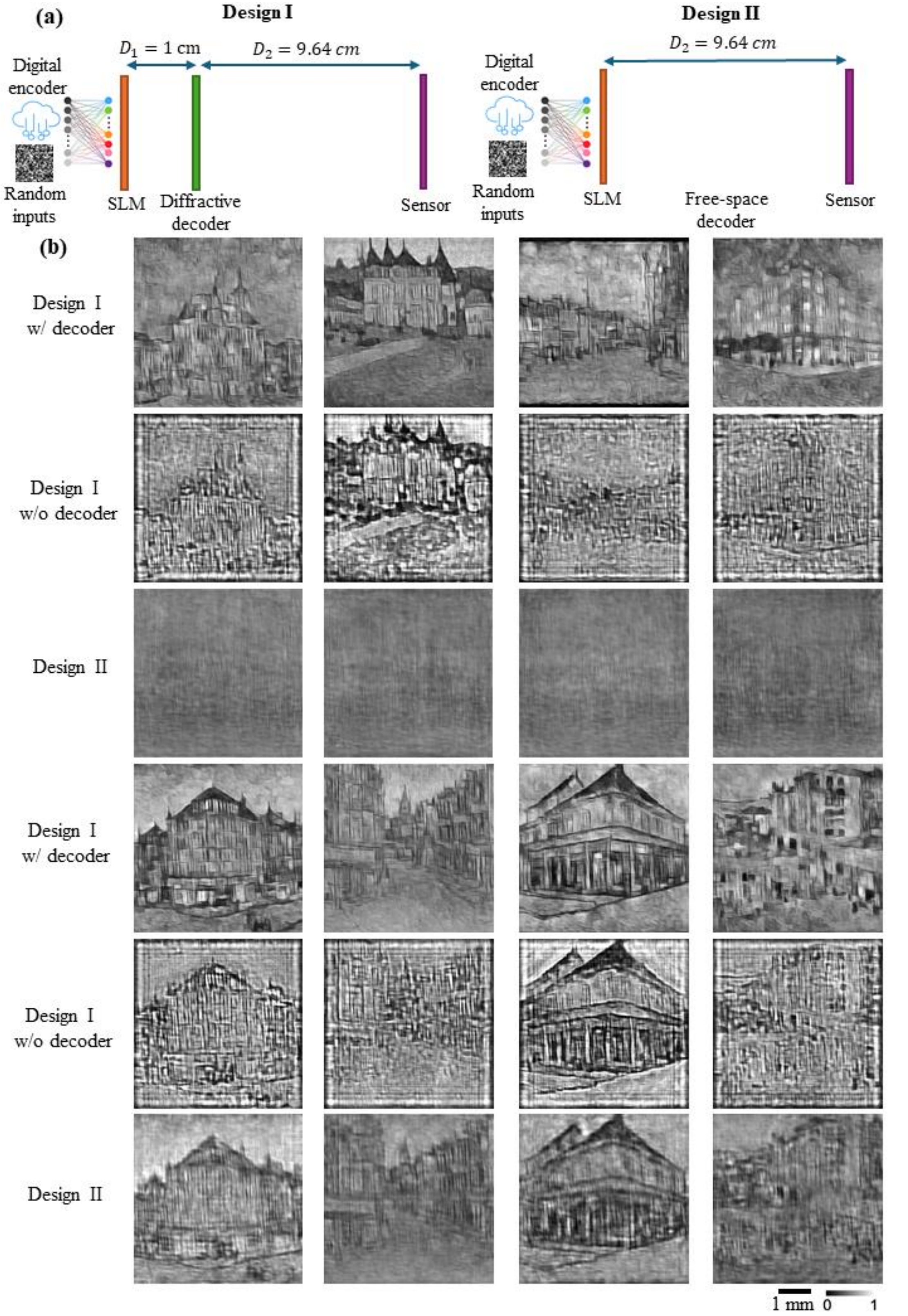

(a) Schematics of Design I trained with the diffractive decoder, and Design II trained directly with the free-space decoder (*i.e.*, without an optimized diffractive layer). The optical

generative model has 85M parameters and D_1 is set to 1cm. (b) Generated images of Design I with or without the diffractive decoder and Design II using the free-space decoder. As shown in the 3rd row, some lower contrast image results fail for the free-space-based decoding, with a CLIP score below 15 empirically considered as a failure. Under this criterion, the failure rate of free-space-based image decoding is 18.8%. Even among the remaining cases, the image generation quality using free-space-based decoding is inferior to that achieved with the jointly optimized diffractive decoder, which is expected due to the additional degrees of trainable parameters available at the diffractive decoder.

Fig. S8: Comparison of diffractive decoder and free-space decoder on Van Gogh style artwork generation.

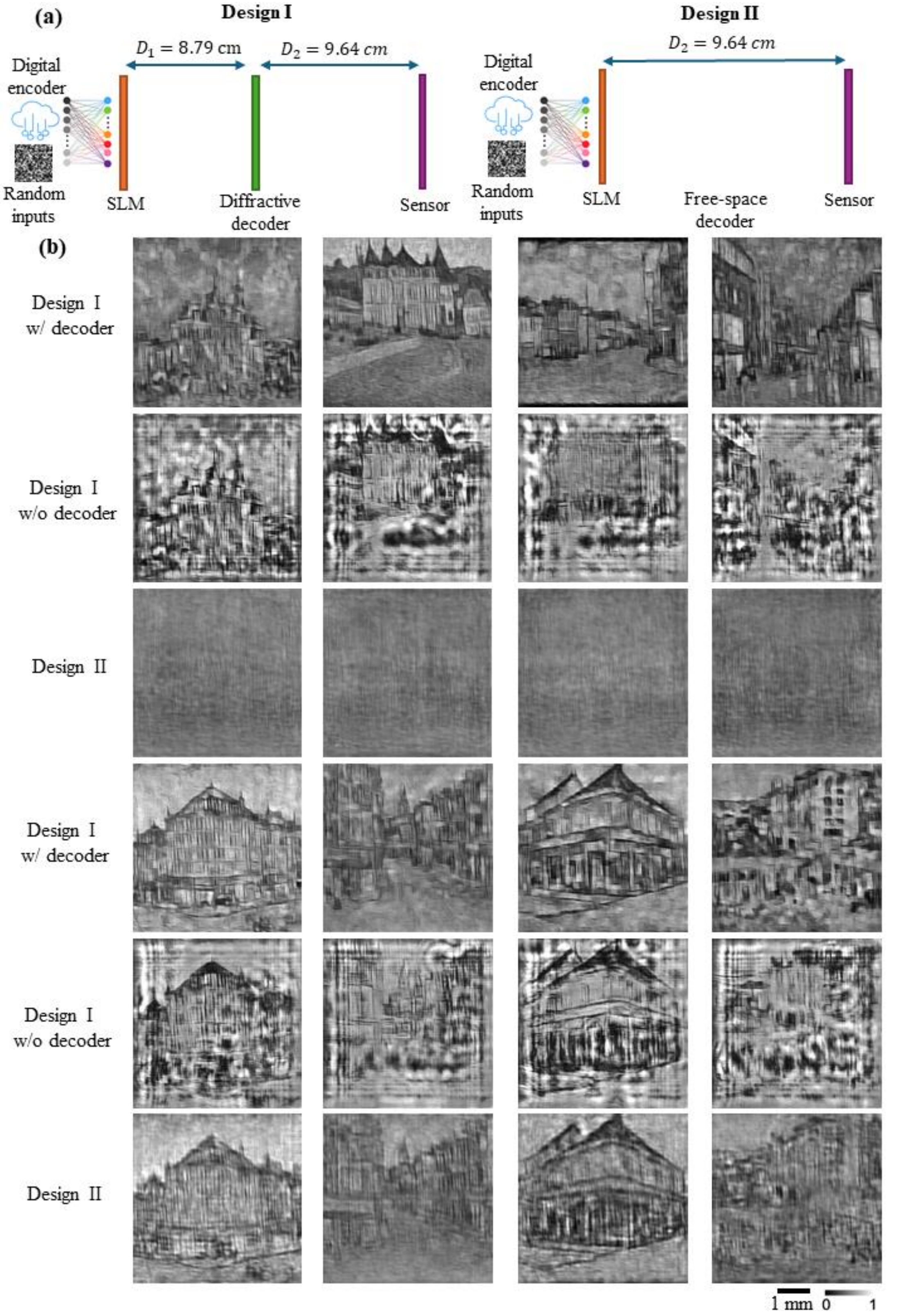

Same as in **Supplementary Fig. S7**, except that D_1 is set to 8.79 cm, simulating the experimental set-up of **Extended Data Fig. 6**. The design with the optimized diffractive layer shows significant performance superiority.

Fig. S9: Numerical and experimental results of a higher resolution snapshot optical generative model for monochrome Van Gogh-style novel artwork generation compared against the teacher digital diffusion model with 1000 steps

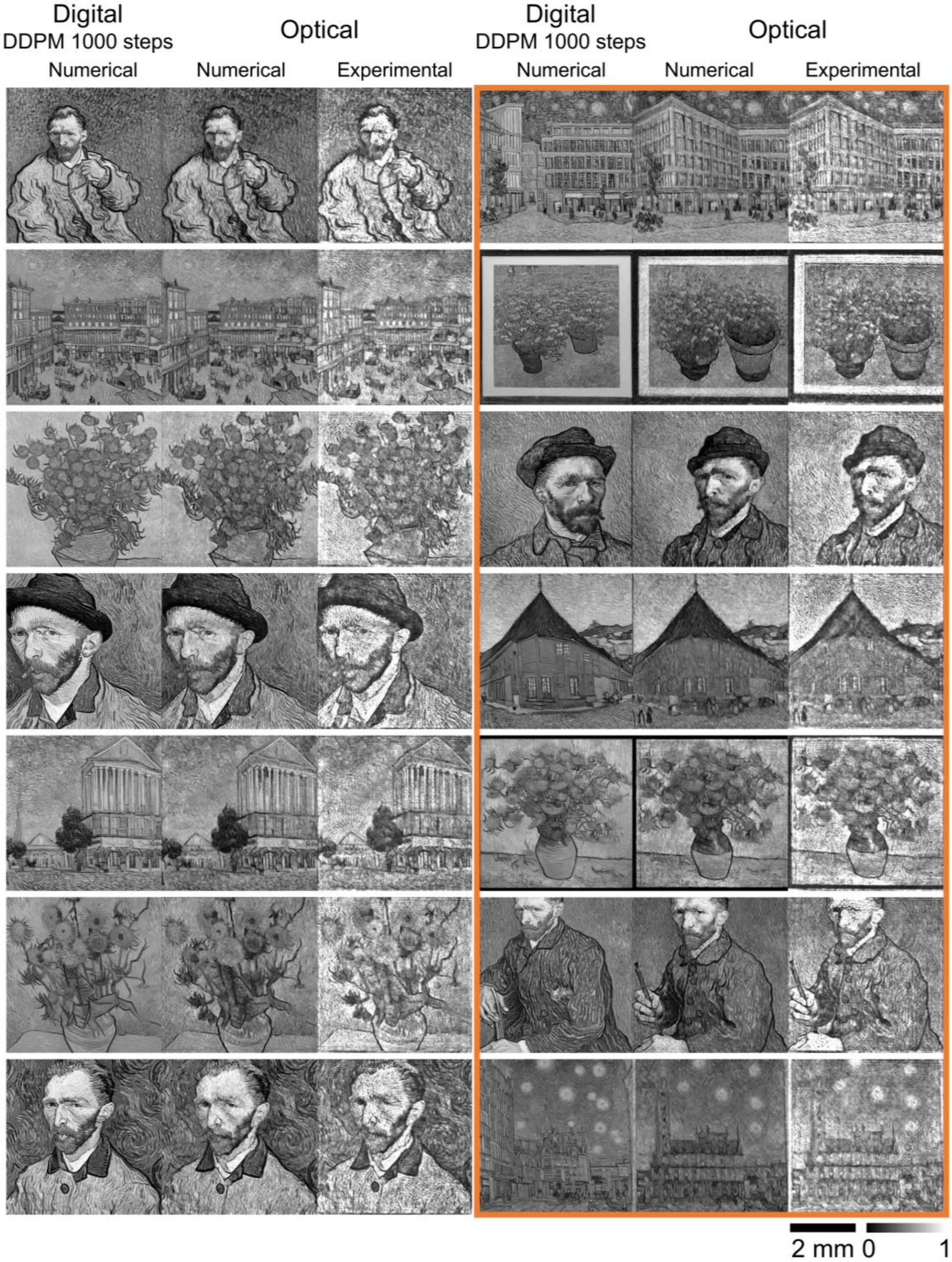

We present comparative results on monochrome Van Gogh-style novel artwork generation for both the digital teacher diffusion model (with 1.07 Billion trainable parameters and 1000 steps used for each inference) and the snapshot optical generative model, along with the experimental results for the snapshot optical generative model. The orange box on the right reveals the discrepancies observed between the digital teacher and the optical model, demonstrating the capability of the snapshot optical generative model to create diverse

images beyond those produced by the digital teacher diffusion model. The digital phase encoder has 580M trainable parameters and each snapshot optical image is generated by a unique random noise input. Input text ("architecture" or "plants" or "person") is used to generate different artworks.

Fig. S10: Numerical and experimental results of a higher resolution snapshot optical generative model for monochrome Van Gogh-style novel artwork generation compared against the teacher digital diffusion model with 1000 steps.

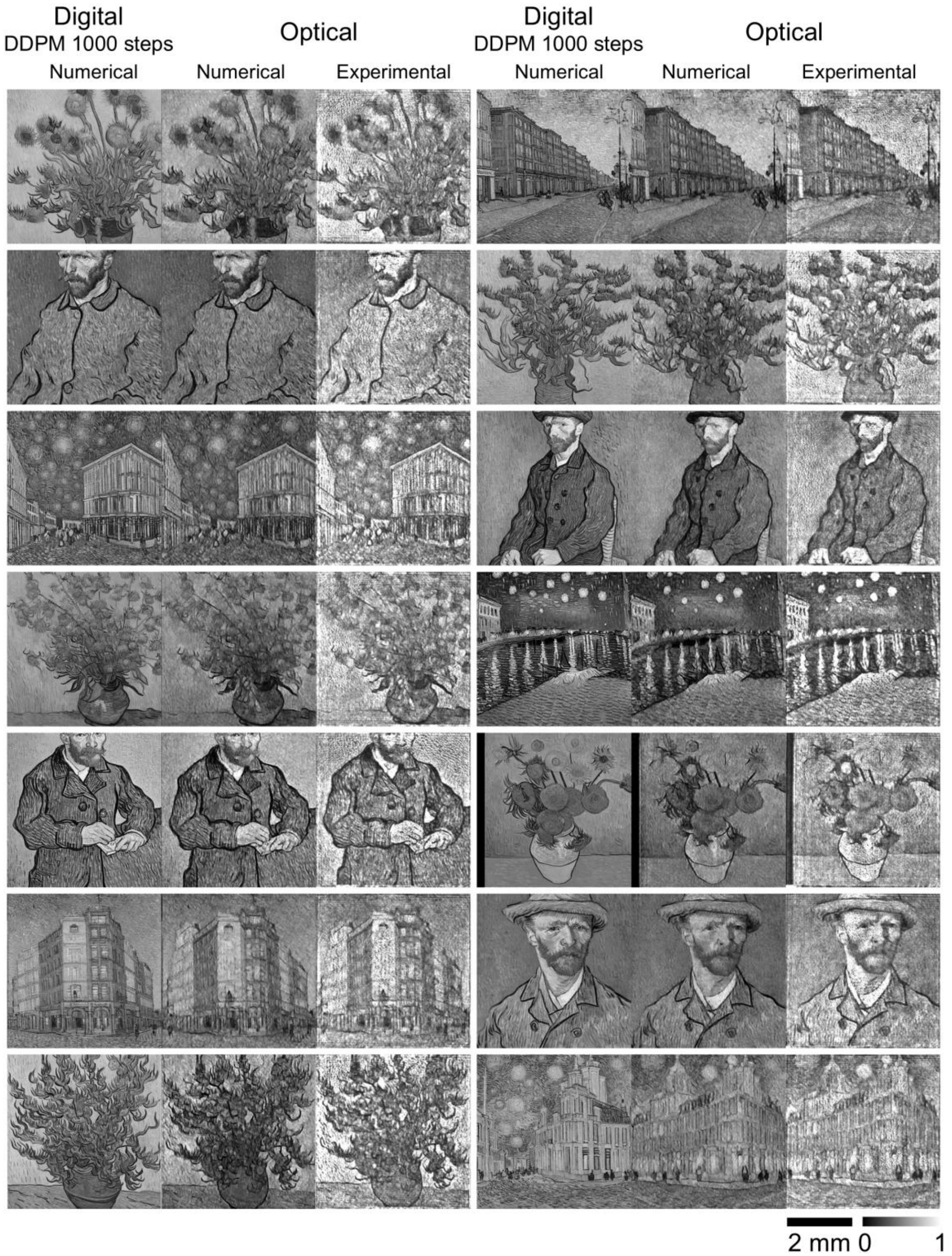

We present comparative results on monochrome Van Gogh-style novel artwork generation for both the digital teacher diffusion model (with 1.07 Billion trainable parameters and 1000 steps used for each inference) and the snapshot optical generative model, along with the experimental results for the snapshot optical generative model. The digital phase encoder has 580M trainable parameters and each snapshot optical image is generated by a unique

random noise input. Input text ("architecture" or "plants" or "person") is used to generate different artworks.

Fig. S11: Numerical and experimental results of a multi-color optical generative model for colorful Van Gogh-style novel artwork generation, compared against the teacher digital diffusion model with 1000 steps.

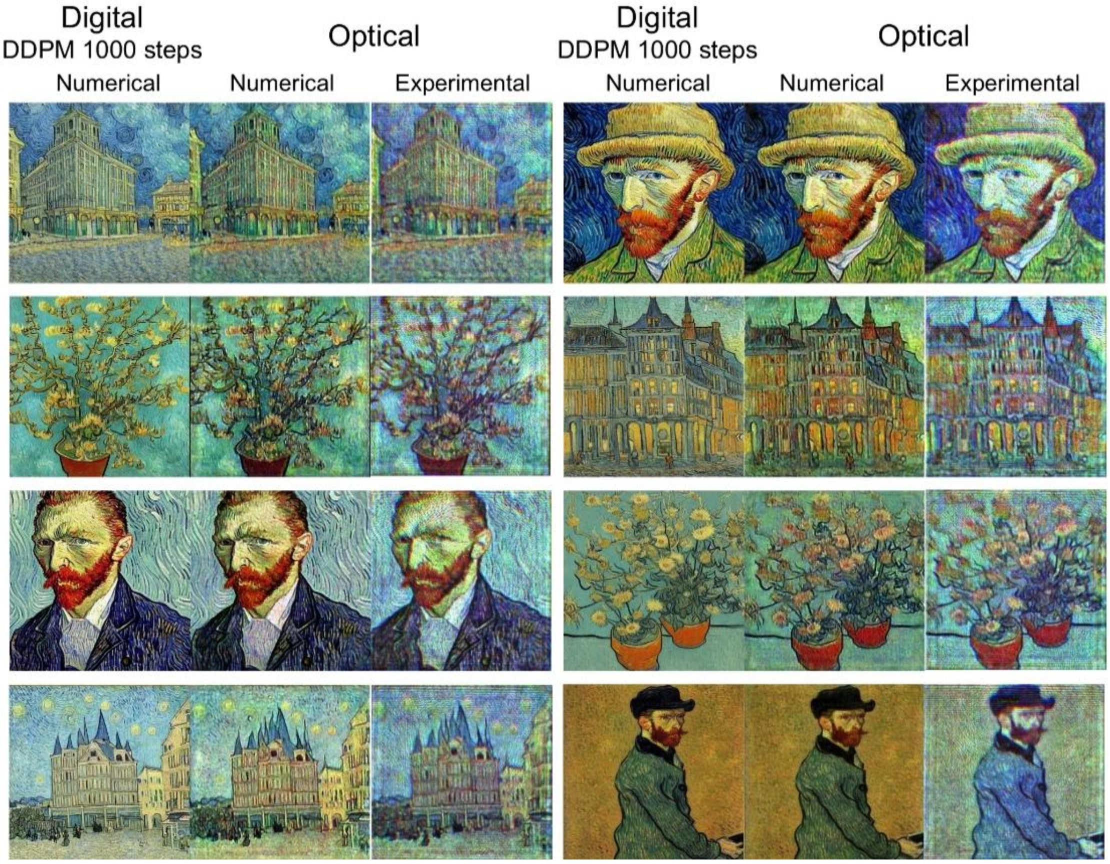

We present numerical and experimental results of a multi-color optical generative model for colorful Van Gogh-style novel artwork generation, compared against the teacher digital diffusion model (with 1.07 Billion trainable parameters and 1000 steps used for each inference). The digital phase encoder has 580M trainable parameters and each snapshot optical image of the RGB channels is generated by a unique random noise input. Input text ("architecture" or "plants" or "person") is used to generate different artworks.

Fig. S12: Numerical and experimental results of a multi-color optical generative model for colorful Van Gogh-style novel artwork generation, compared against the teacher digital diffusion model with 1000 steps.

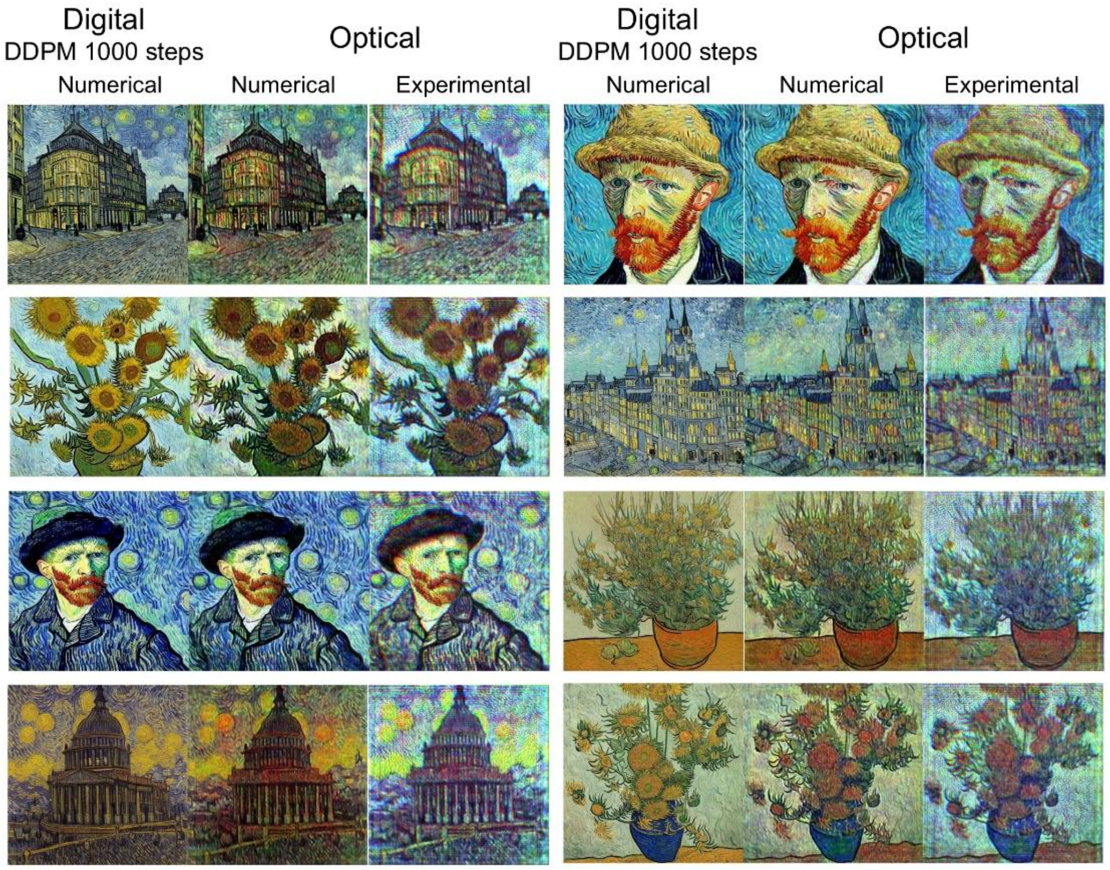

We present numerical and experimental results of a multi-color optical generative model for colorful Van Gogh-style novel artwork generation, compared against the teacher digital diffusion model (with 1.07 Billion trainable parameters and 1000 steps used for each inference). The digital phase encoder has 580M trainable parameters and each snapshot optical image of the RGB channels is generated by a unique random noise input. Input text ("architecture" or "plants" or "person") is used to generate different artworks.

Fig. S13: Fidelity comparisons between the numerical and experimental results of the optical generative model.

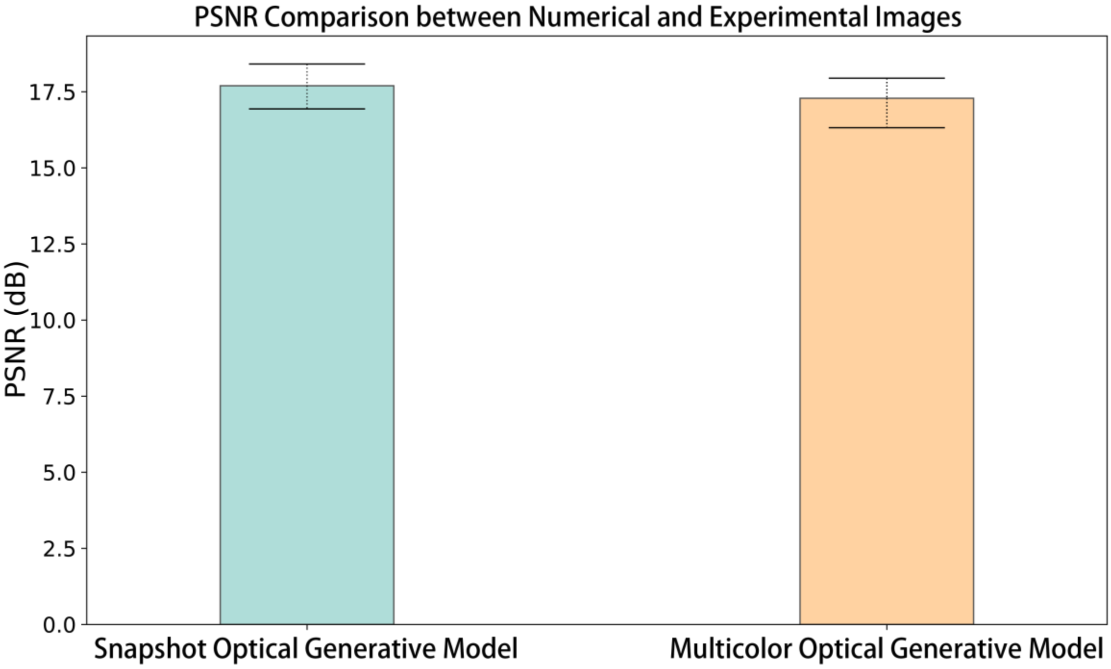

We report the PSNR values calculated between the numerically simulated and experimentally generated results. For the snapshot optical generative model, the results correspond to **Fig. 4**, **Supplementary Fig. S9**, and **Supplementary Fig. S10**. For the multicolor optical generative model, the evaluations correspond to the results from **Fig. 5**, **Supplementary Fig. S11**, and **Supplementary Fig. S12**.

Fig. S14: CLIP score evaluation of the text-to-image alignment for Van Gogh style artwork generation.

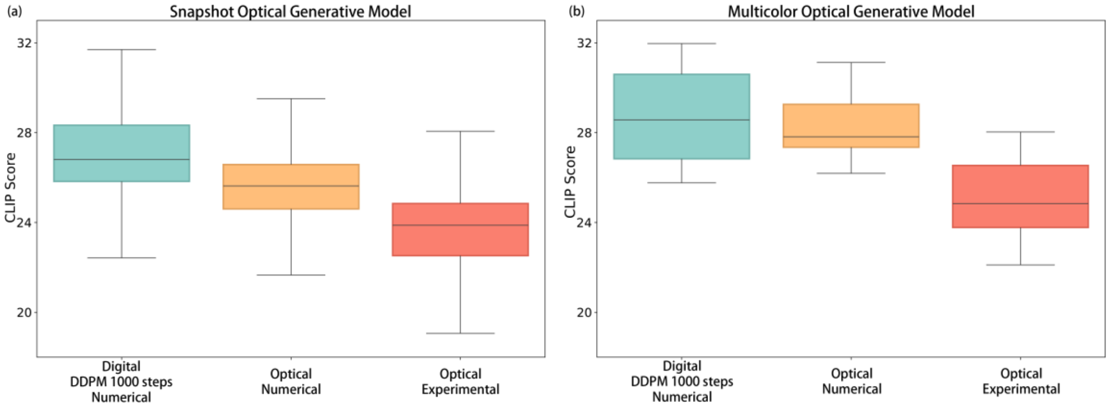

We present the contrastive language-image pre-training score (CLIP score[4]) for both the numerical and experimental results of the Optical Generative Model, compared against the teacher digital diffusion model (with 1.07 Billion trainable parameters and 1000 steps used for each image inference). The CLIP score quantifies the semantic alignment between the generated images and the reference text: "*Van Gogh style painting of {architecture, plants, person}*". The CLIP score evaluation for the snapshot optical generative model (left) corresponds to **Fig. 4**, **Supplementary Fig. S9**, and **Supplementary Fig. S10**. The evaluation for the multicolor optical generative model (right) corresponds to **Fig. 5**, **Supplementary Fig. S11**, and **Supplementary Fig. S12**.

Fig. S15: The impact of limited phase modulation levels on snapshot optical generative models.

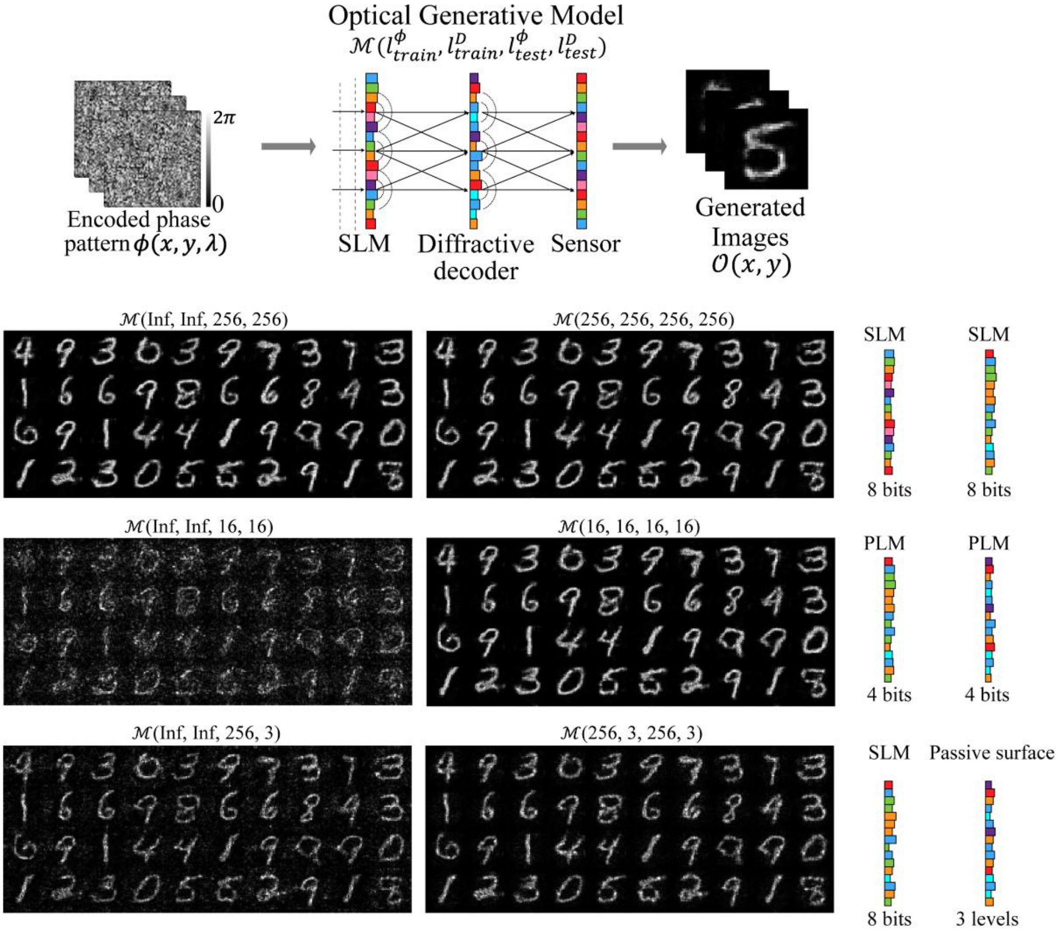

$l^{\phi}_{train}, l^{D}_{train}, l^{\phi}_{test}, l^{D}_{test}$ represent the discrete phase modulation levels of the SLM and the diffractive decoder during the training ($train$) and testing ($test$), respectively. The blind testing performance can be improved significantly by including the modulation bit depth limitation of the snapshot optical generative model hardware during the training process. $M(l^{\phi}_{train}, l^{D}_{train}, l^{\phi}_{test}, l^{D}_{test})$ refers to the snapshot optical generation model trained and tested under $l^{\phi}_{train}, l^{D}_{train}, l^{\phi}_{test}, l^{D}_{test}$.

Fig. S16: Determination of image generation failures using noise variance.

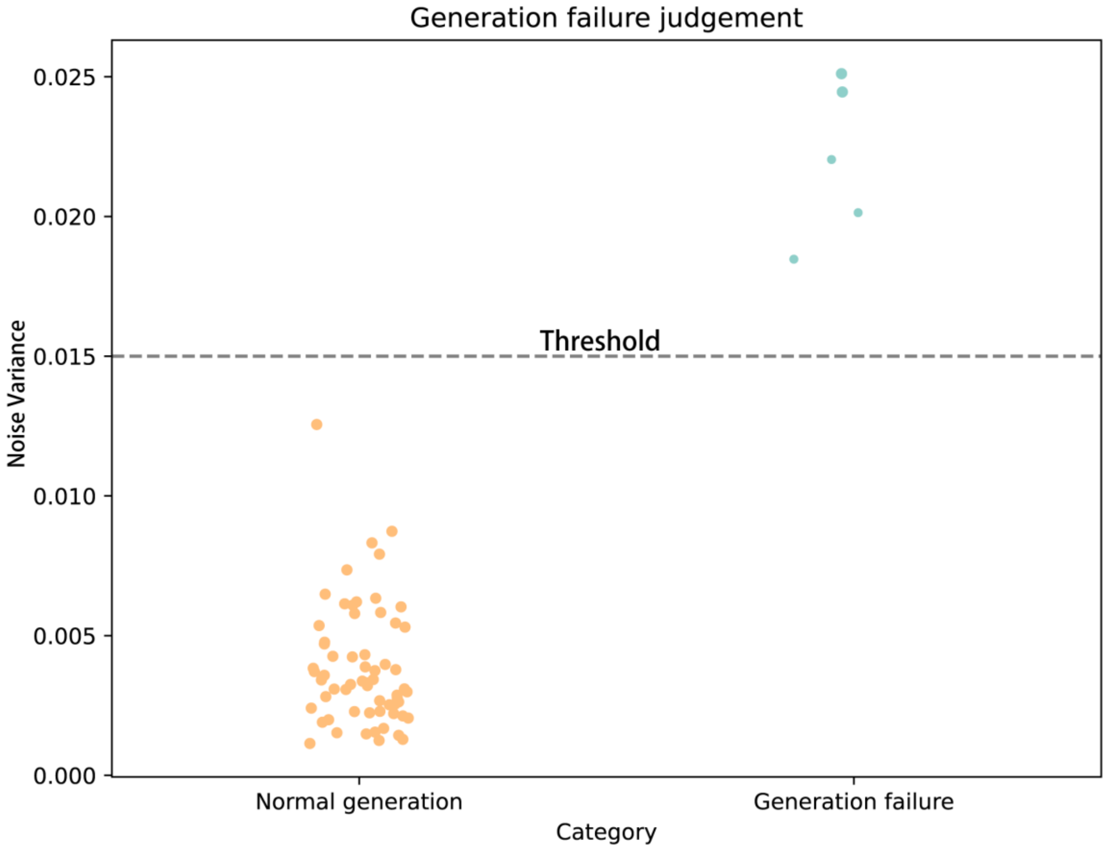

Generated images with noise variance higher than a predefined threshold (0.015) are considered as image generation failures.

Fig. S17: Detailed analysis of optical generative models with different features and training methods.

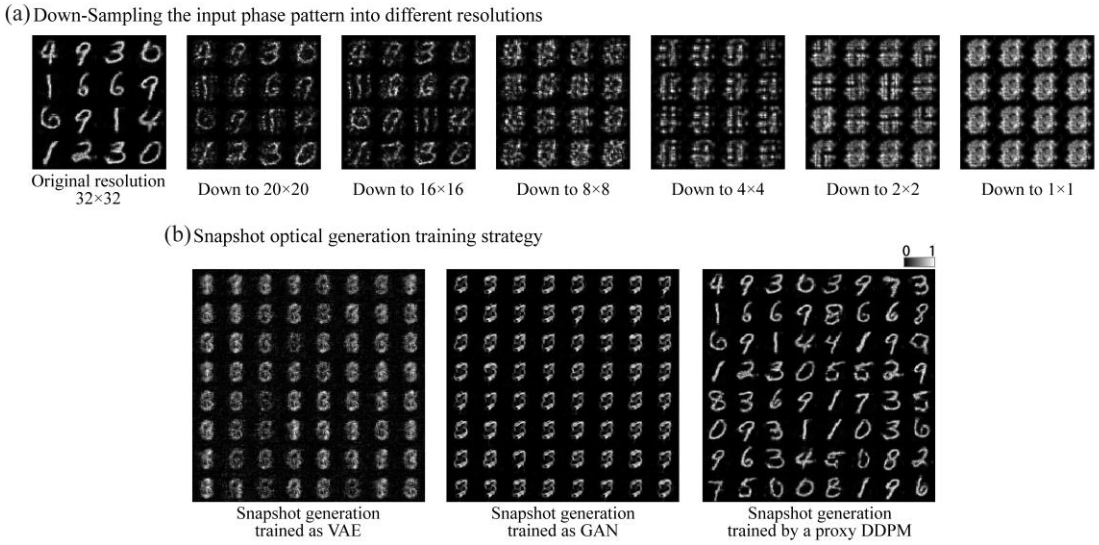

(a) Impact of the resolution of the phase-encoded optical generative seed patterns and the novel image generation performance. (b) Different training strategies for snapshot optical generative models are compared.

Fig. S18: Performance comparison of snapshot optical generative models against digital-only GAN-based generative models trained on MNIST dataset.

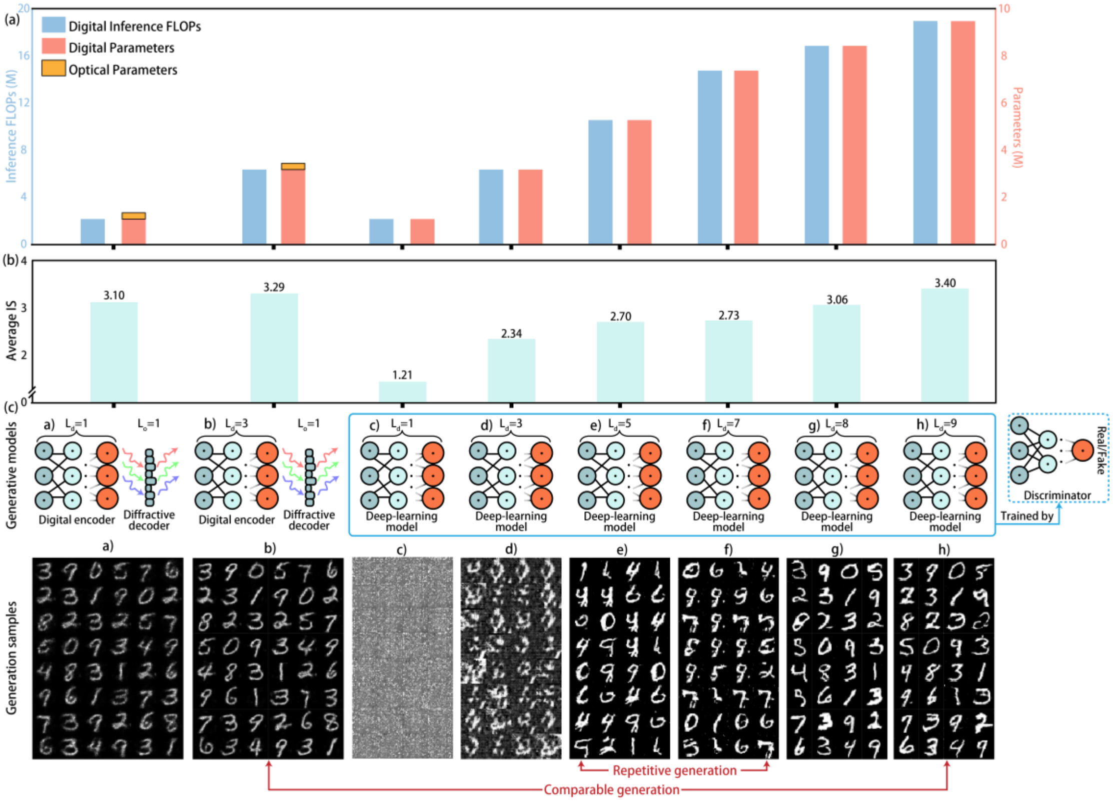

(a) Inference FLOPs and the number of trainable parameters for different models used in the MNIST image generation. (b) Average IS values of the corresponding models. (c) Generative model structures and image generation samples: a) and b) represent the snapshot optical generative models; c) to h) correspond to GAN-based digital generative models. L_d and L_o stand for the number of FC digital layers and the number of diffractive decoder layers, respectively. The image generation results from c) to f) show that generative models with fewer parameters face difficulty in directly capturing the data distribution in a generative adversarial learning scheme.

Fig. S19: Performance comparison of MNIST snapshot optical generative models against digital-only generative models guided by diffusion.

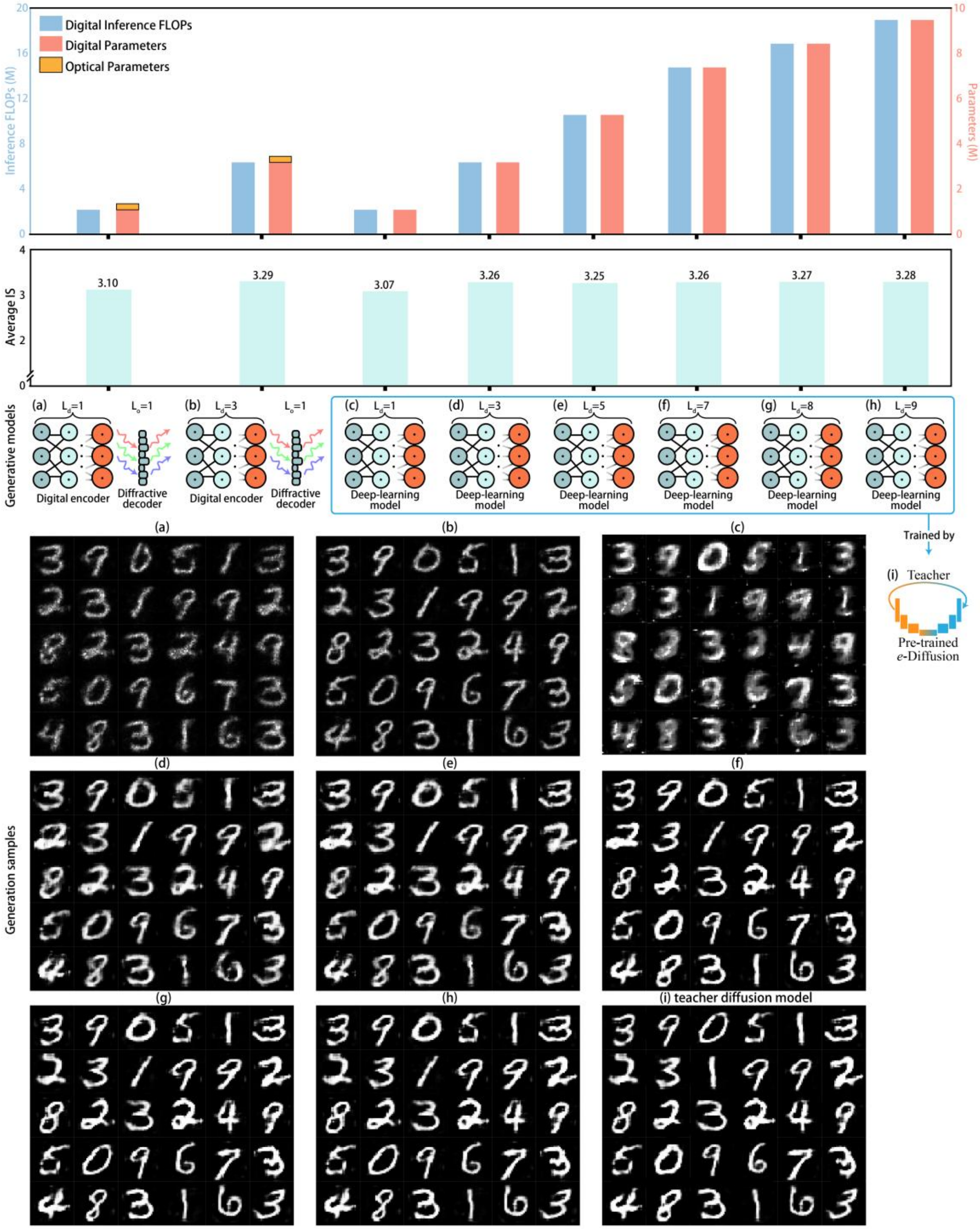

(a) Inference FLOPs and the number of trainable parameters of different models for MNIST image generation. (b) Average IS values of the corresponding models. (c) Generative model structures and image generation samples: a) and b) represent snapshot optical generative models; c) to h) correspond to diffusion-teacher-guided digital generative models; i) represents a pre-trained digital diffusion-based generative model. L_d and L_o stand for the number of FC digital layers and the number of diffractive decoder layers, respectively.

Fig. S20: Performance comparison of Fashion-MNIST snapshot optical generative models against digital-only generative models guided by diffusion.

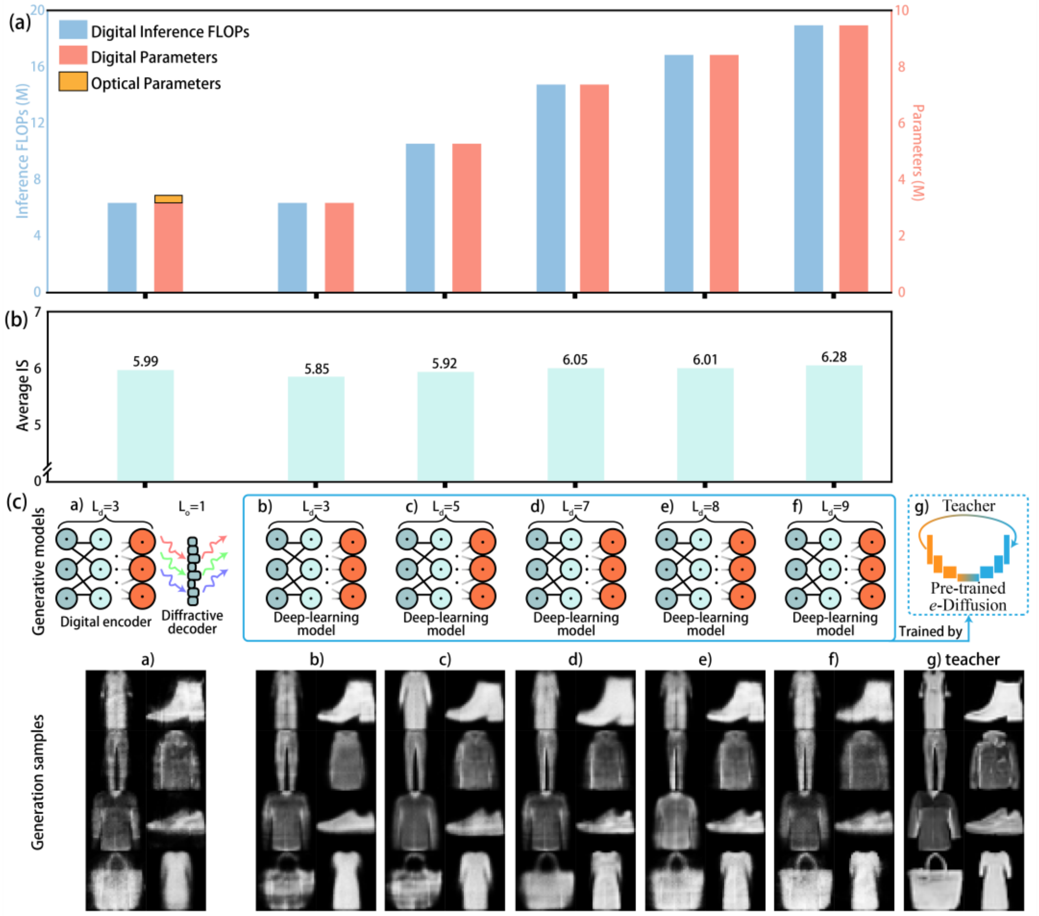

(a) Inference FLOPs and the number of trainable parameters of different models for Fashion-MNIST image generation. (b) Average IS values of the corresponding models. (c) Generation model structures and image generation samples: a) represents snapshot optical generative models; b) to f) correspond to diffusion-teacher-guided digital generative models; g) represents a pretrained digital diffusion generative model. L_d and L_o stand for the number of FC digital layers and the number of diffractive decoder layers, respectively.

Fig. S21: Performance comparison of MNIST snapshot optical generative models against DDPM-based generative models

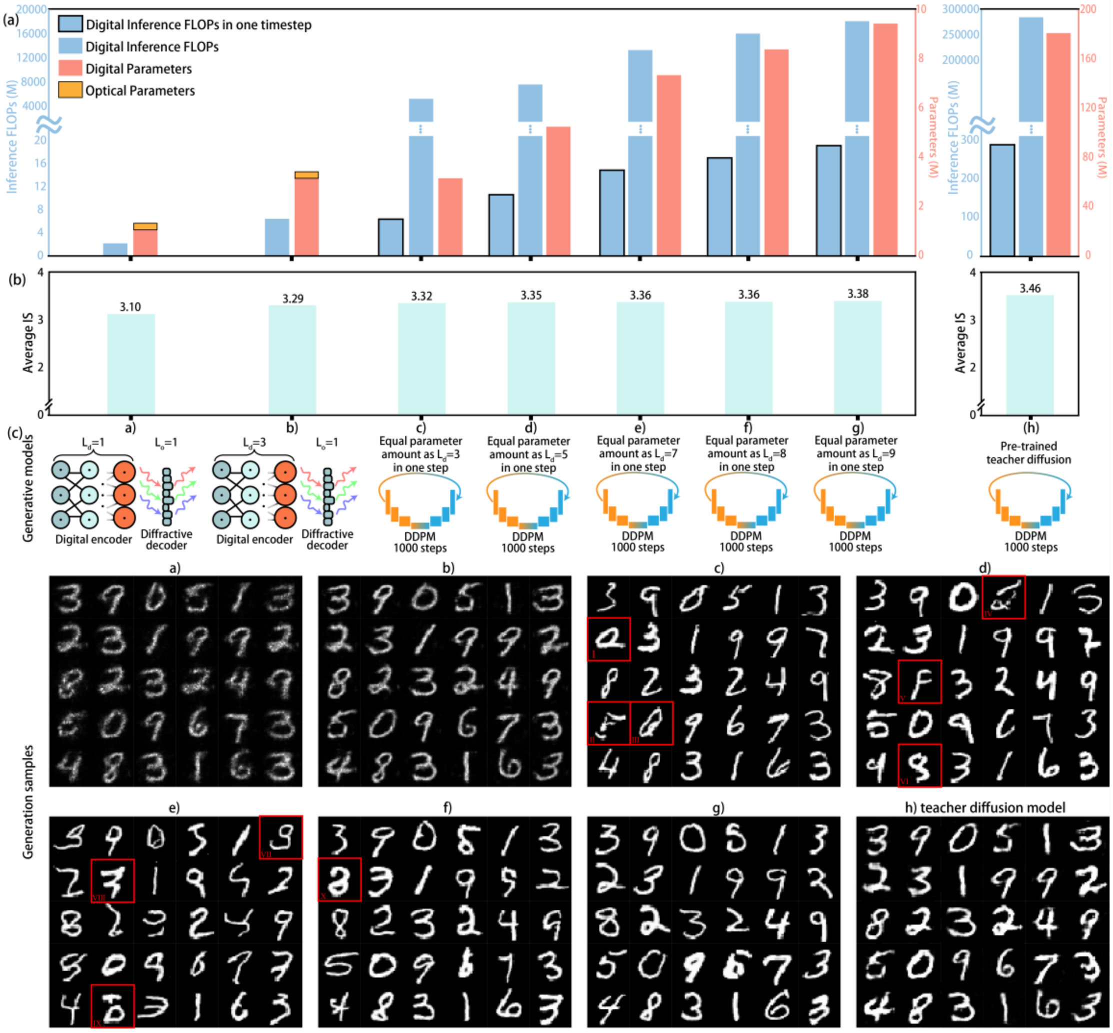

(a) Inference FLOPs and the number of trainable parameters of different models for MNIST image generation. (b) Average IS values of the corresponding models. (c) Generation model structures and image generation samples: a) and b) are snapshot optical generative models, c) to g) correspond to DDPM with 1000-step image generation. The U-Net in DDPM shown in each case, **c) to g)**, has an equal number of trainable parameters as the digital encoders in **Fig. S18 d) to h)**, respectively; h) on the right represents a pre-trained digital diffusion model-based image generation, also using 100 steps. The generated images shown within the red boxes represent some of the unsuccessful digital generations of the corresponding DDPM, sequentially conditioned on the labels: {2, 5, 0, 5, 2, 8, 3, 3, 8, 2} (from Red Box I to X, respectively).

Fig. S22: Ablation study on the key components of snapshot optical generative models.

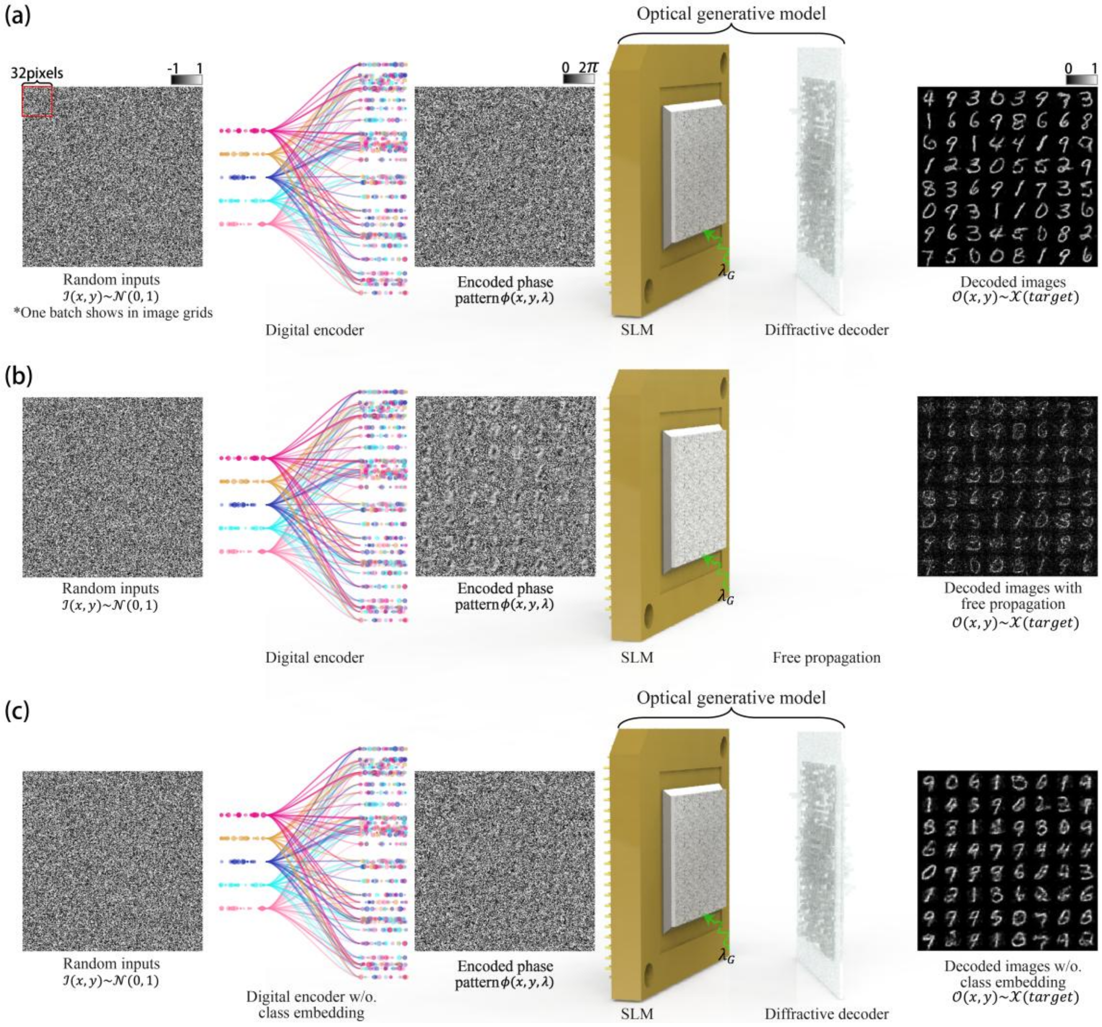

(a) Snapshot optical generative model. (b) Ablation study on the diffractive decoder (replaced by free-space propagation). (c) Ablation study on the class embedding in the digital encoder, *i.e.*, the class embedding is eliminated.

Fig. S23: Performance investigation of snapshot optical generative models as a function of the SLM phase range.

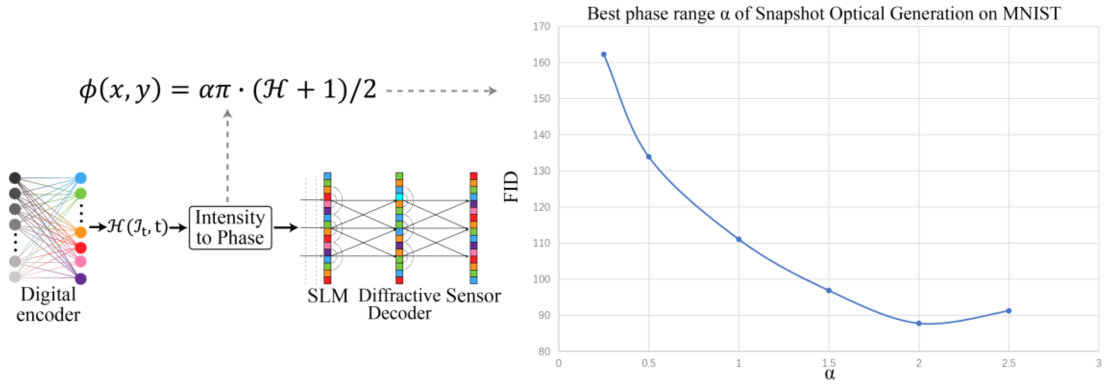

Relationship between the phase range α and the novel image generation quality of a snapshot optical generative model.

Fig. S24: Performance evaluation of iterative optical generative models with different depths of the diffractive decoder

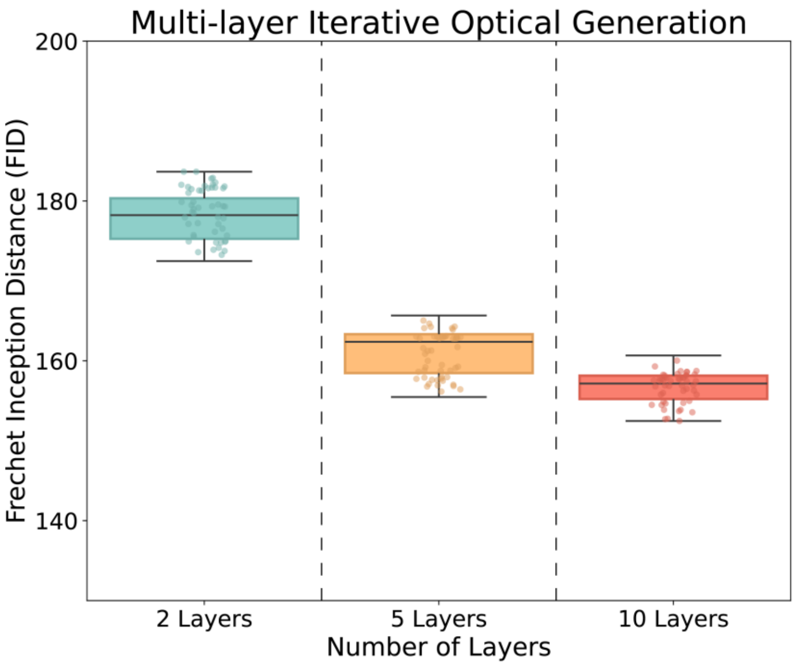

Image generation ability of an iterative optical generative model with a multi-layer diffractive decoder ($L_o = 2, 5, 10$ layers), which shows the scalability of the diffractive decoder architecture.

Fig. S25: Performance investigation of iterative optical generative models as a function of misalignments.

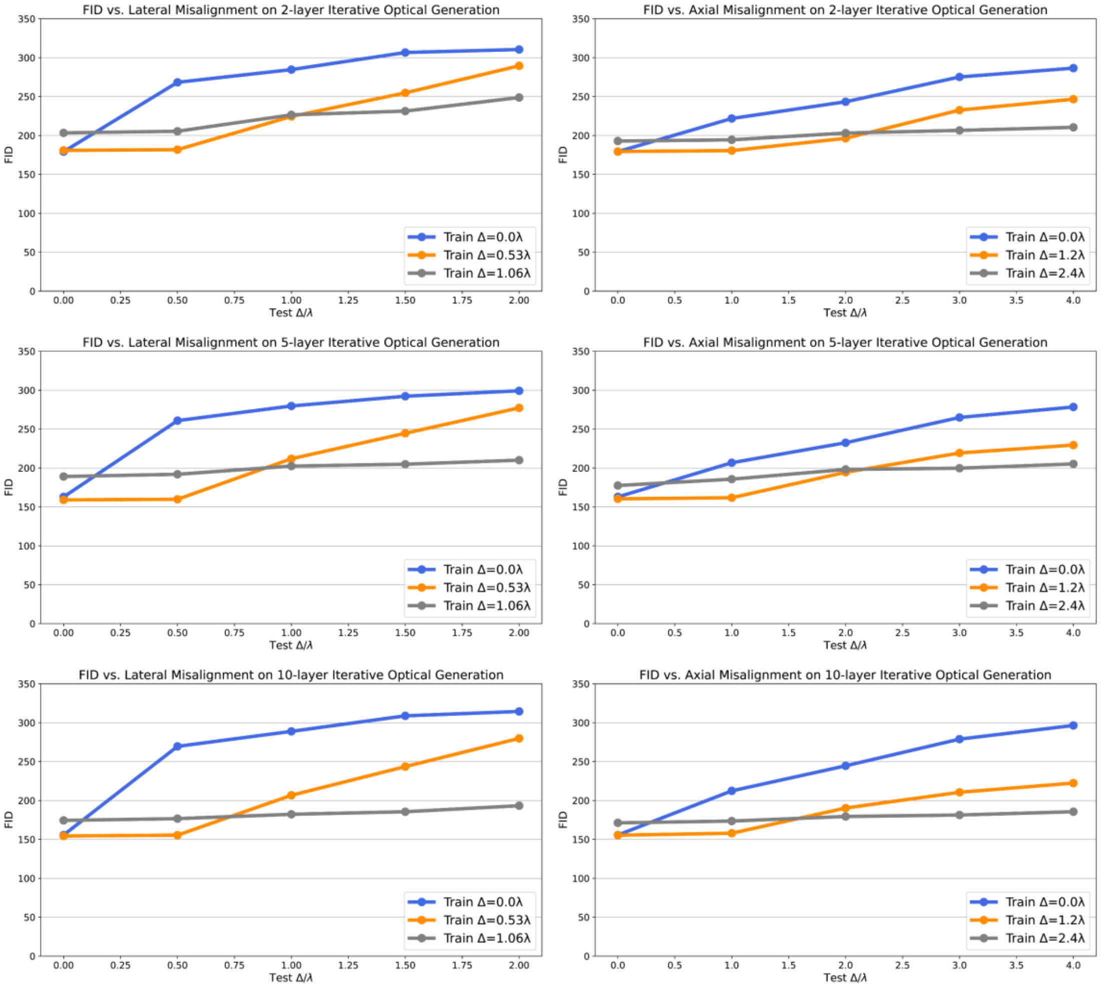

Misalignment evaluation of an iterative optical generative model with a multi-layer diffractive decoder ($L_o = 2, 5, 10$ layers). In the left part, the blue, orange, and gray curves represent the misalignment robustness evaluation of the optical generative models trained with random lateral misalignments (along both x and y directions) in the range of 0.0, ±0.53, and ±1.06λ, respectively. On the right, the same analysis focuses on axial misalignments along the z direction. Training the iterative optical generative model with small amounts of random disturbances/misalignments makes the blind inference more robust against such unknown, random perturbations, as shown with the gray curves.

Fig. S26: Experimental results of the snapshot optical generative model using interpolated latent noises

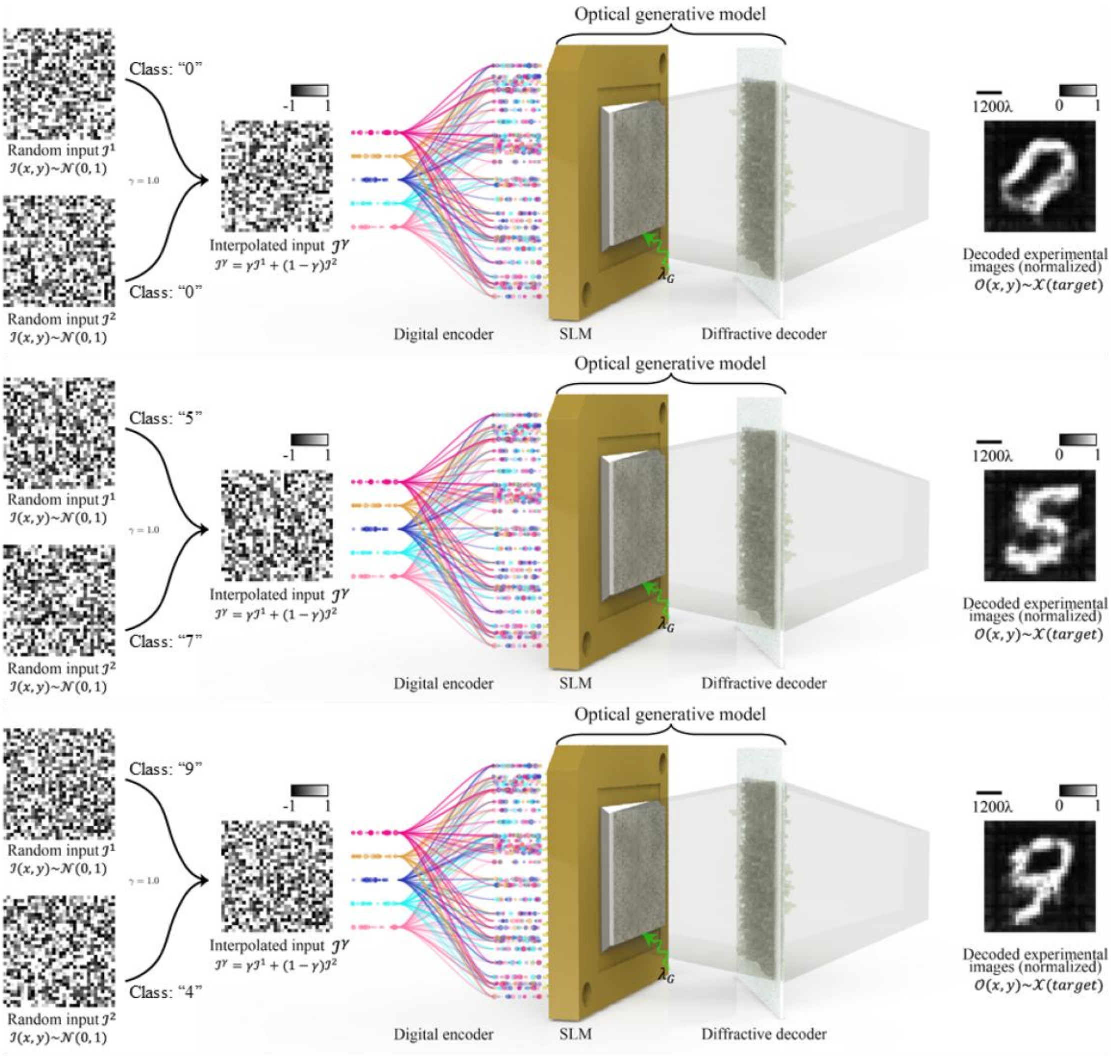

Refer to **Supplementary Videos 3-9** for additional details. Also see **Extended Data Fig. 5** of the main text, which shows how the process of latent interpolation is controlled by the weights along with the interpolated class embeddings, gradually transforming the generated images from one digit to another.

Fig. S27: U-Net model architecture used in digital DDPM

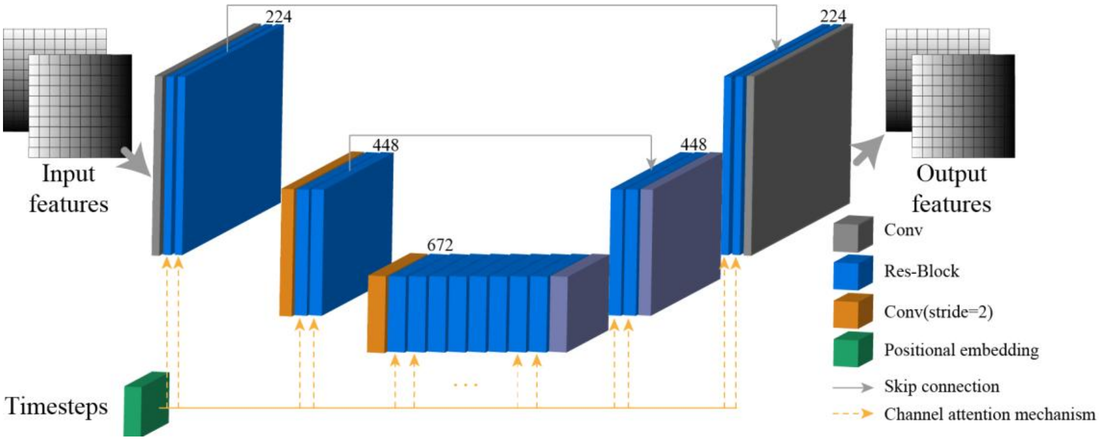

Details of the U-Net model used in the proxy DDPM. The channel dimensions of each block are also listed.

References

1. He, K., Zhang, X., Ren, S. and Sun, J., 2016. Deep residual learning for image recognition. In *Proceedings of the IEEE conference on computer vision and pattern recognition* (pp. 770-778).
2. Ho, J., Jain, A. and Abbeel, P., 2020. Denoising diffusion probabilistic models. *Advances in neural information processing systems*, *33*, pp.6840-6851.
3. Xu, B., 2015. Empirical evaluation of rectified activations in convolutional network. *arXiv preprint arXiv:1505.00853*.
4. Hessel, Jack, et al. "CLIPScore: A Reference-free Evaluation Metric for Image Captioning." *Proceedings of the 2021 Conference on Empirical Methods in Natural Language Processing (EMNLP)*, 2021.
